# Elucidating the solution structure of the monomolecular *BCL2* RNA G-quadruplex: a new robust NMR assignment approach†

**DOI:** 10.1039/d5sc01416f

**Published:** 2025-03-26

**Authors:** Zenghui Wang, Carla Ferreira Rodrigues, Simon Jurt, Alicia Domínguez-Martín, Silke Johannsen, Roland K. O. Sigel

**Affiliations:** a Department of Chemistry, University of Zürich 8057 Zürich Switzerland silke.johannsen@chem.uzh.ch roland.sigel@chem.uzh.ch; b Department of Inorganic Chemistry, Faculty of Pharmacy, University of Granada 18071 Granada Spain

## Abstract

5′ untranslated regions (UTRs) of mRNA commonly feature G-quadruplexes (G4s), crucial for translational regulation and promising as drug targets to modulate gene expression. While NMR spectroscopy is well-suited for studying these motifs' structure and dynamics, their guanine-rich nature complicates resonance assignment due to high signal overlap. Exploiting the inherent rigidity of G4 cores, we developed a universally applicable assignment strategy for uniformly isotopically enriched G4 structures, relying solely on through-bond correlations to establish the G-tetrads. Applying this approach, we resolved the solution structures of two triple mutants of the RNA G4 in the 5′ UTR of the human *BCL2* proto-oncogene, one of the first natural monomolecular RNA G4 structures available to date. Comparative analysis with other RNA and DNA G4s reveals their notably compact and well-defined cores. Moreover, the sugar pucker geometries of the tetrad guanines are far less stringent than previously assumed, adeptly accommodating specific structural features. This contrasts with the canonical base pairing in RNA and DNA, in which the sugar pucker dictates the type of the double-helical structure. The strategy presented provides a direct path to uncovering G4 structural intricacies, advancing our grasp of their biological roles, and paving the way for RNA-targeted therapeutics.

## Abstract 2

A novel assignment strategy for RNA G-quadruplexes and their structure determination using the *BCL2* sequence is presented.

## Graphical abstract

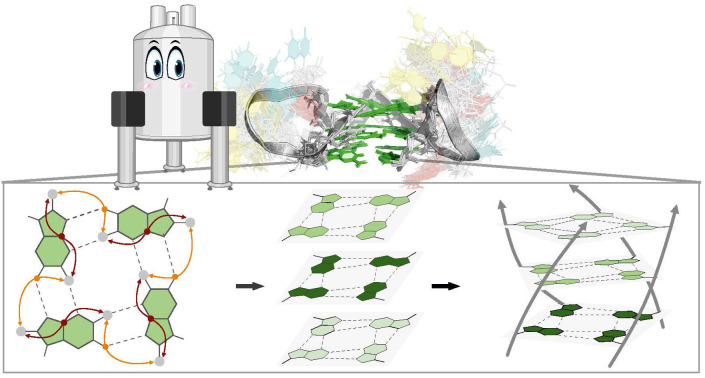

## Introduction

The discovery of G-quadruplexes (G4) in guanine-rich DNA and RNA sequences in 1987^1,2^ constituted a revolutionary advance in nucleic acid research, opening up a new field of study into the structural diversity and their individual functional roles.^3–6^ Originally identified in telomeres, G4 structures have since been found throughout the human genome, with over 700 000 putative DNA G4 structures reported.^7^ Despite extensive *in vitro* studies, the *in vivo* existence of DNA and RNA G4 structures was not confirmed until 2014.^8–10^ While initial research focused mainly on DNA G4s, recent advancements have revealed the pivotal roles of mRNA G4s in regulating translation, mRNA processing, transcription termination, mRNA localisation, and alternative splicing.^11–14^ Over 13 000 putative RNA G4 sequences have been identified in the human transcriptome^15^ of which 500 are in 5′ untranslated regions (5′ UTRs), impacting the translation efficiency of key proteins such as NRAS,^16^ human VEGF,^17,18^ TGFβ2,^19^ and Bcl-2.^20^

**Fig. 1:**
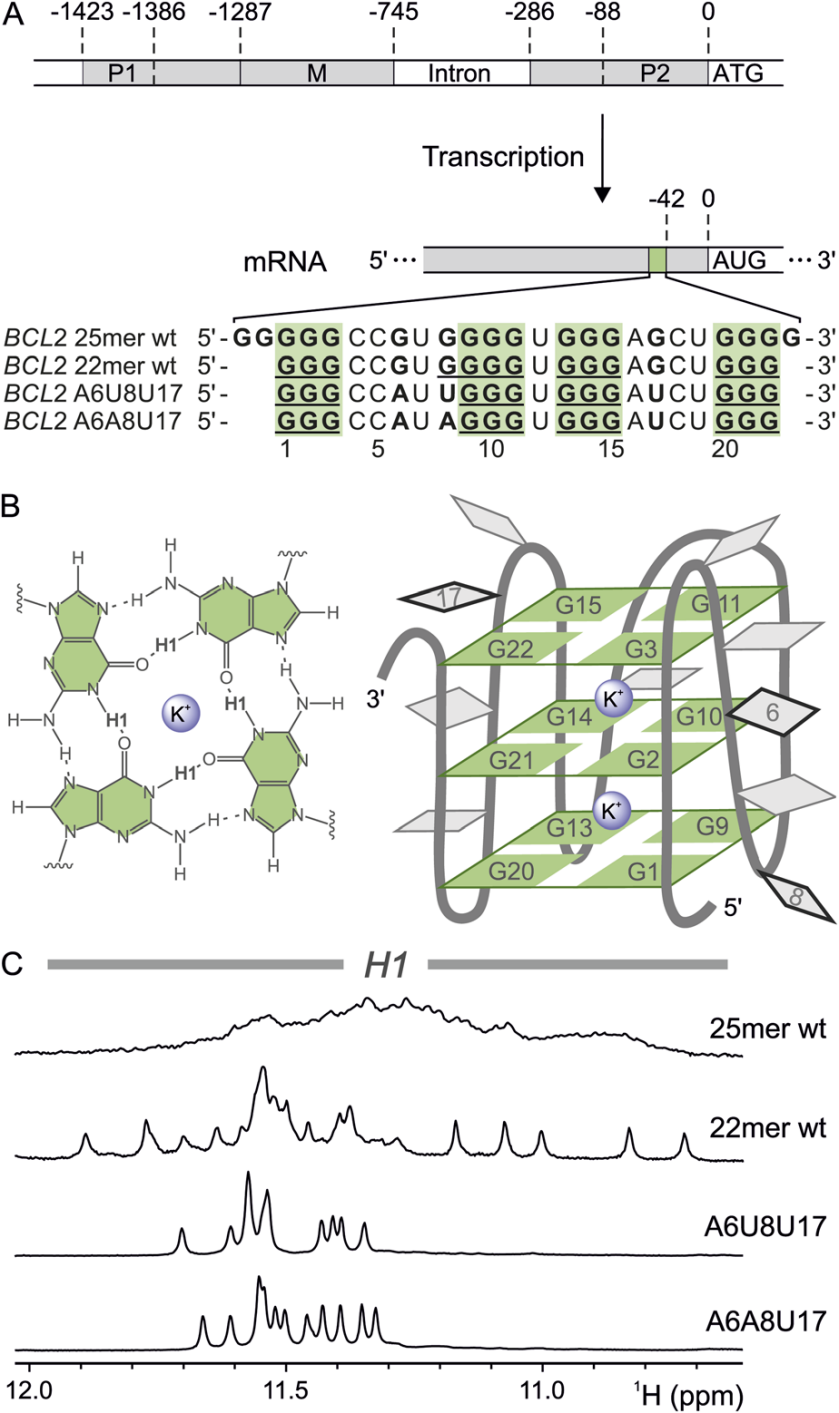
The *BCL2* G-quadruplex. (A) Schematic representation of the *BCL2* DNA sequence with the promoter regions P1, P2, and M. Exons are depicted in light grey, and introns are white. The *BCL2* mRNA with the 5′ UTR G4 (light green), located 42 nucleotides upstream of the AUG start codon, is shown below. In addition, the sequences of the 25mer wt, the truncated 22mer wt and the two *BCL2* G4 mutants A6A8U17 and A6U8U17 are shown. (B) Schematic representation of a G4 tetrad (left) and the G4 structure with sequence numbering of the core guanines and the mutated nucleotides (right). The nucleotides in the loops are depicted in grey, and the positions of the mutated nucleotides are framed in black. Metal ions are represented as purple spheres. (C) H1 region of the ^1^H NMR spectra (H_2_O/D_2_O (9 : 1), 2 mM KCl, pH 7, 298 K) of the different *BCL2* G4 sequences, 25mer wt (0.1 mM), 22mer wt (0.4 mM) and the two triple mutants A6U8U17 (1 mM) and A6A8U17 (1.3 mM).

G4s consist of a rigid core and flexible interconnecting loops, as shown in Fig. 1B. The core is formed by π–π stacking of tetrads, each a coplanar cyclic arrangement of four Hoogsteen-paired guanines. The core is additionally stabilised by metal ions coordinating the partially negatively charged carbonyl groups of the guanines in the tetrad, thereby forming a metal ion channel in the centre.^21^ In particular, monovalent metal ions have a high stabilising effect, with potassium(i) having the highest stabilisation energy.^22,23^ Previously, RNA G4s were believed to be strictly parallel, with all four strands pointing in the same direction. Recent studies, however, have shown synthetic RNA G4 structures with antiparallel and hybrid connectivity,^24–27^ formerly ascribed only to DNA G4s.^28^ The guanine-rich regions frequently possess excess guanines beyond the requisite core formation, fostering highly dynamic systems in which guanines within the loops exchange with guanines in the core.^29^ This dynamic interplay of structures potentially plays a pivotal role in their regulatory function, but at the same time challenges structure elucidation.

To date, roughly 500 G4 structures have been determined, of which around 90 are RNA G4s.^30,31^ These RNA structures were elucidated with X-ray crystallography and NMR (nuclear magnetic resonance) spectroscopy, and recently, the first Cryo-EM structure of a synthetic aptamer sequence was released.^32^ Most determined RNA G4 structures are multi-molecular G4s,^30,33^ RNA aptamers or other synthetic constructs bound to proteins or ligands.^26,32,34–46^ Less than 10 of 90 determined structures are naturally occurring monomolecular G4s, one of which interacts with a large protein complex,^47^ while the others are stand-alone RNA G4s.^48–51^

The relatively small size of G4s (around 20–30 nt) makes NMR spectroscopy an ideal method for determining their structures and analysing their dynamics over different time scales.^52,53^ NMR structure determination of nucleic acids relies heavily on assigning ^1^H resonances and their through-space correlations.^53,54^ In G4 structures, however, the high guanine content leads to low signal dispersion. Furthermore, their dynamic character can cause severe line broadening and increased spectral overlap due to conformational polymorphism, depending on the time scale, complicating chemical shift assignment. For DNA G4s, this challenge is typically resolved through site-specific incorporation of isotope-enriched nucleotides *via* solid-phase synthesis until complete assignment is achieved.^55^ The corresponding isotope-enriched ribo-phosphoramidites have only been available for a few years, preventing earlier application of this respective strategy.^56^ Nowadays, generating multiple RNA constructs for unambiguous assignment *via* solid-phase synthesis is more feasible, if still time-consuming, costly, laborious and highly dependent on the length of the studied RNA. *In vitro* transcription is a one-pot, length-independent, alternative method that provides high yields of *in vitro* folded RNA^57^ and the ability to insert a wide range of isotopically enriched or otherwise modified nucleotides in a nucleotide-uniform manner. However, NMR structural elucidation of G4s from *in vitro* transcribed RNA still lacks a robust assignment strategy to manage the high signal density and overlap effectively.

In this study, we present the NMR structure determination of two triple mutants (A6A8U17 and A6U8U17) of the monomolecular *BCL2* RNA G4 structure, effectively capturing this G4 in a singular conformation. Employing *in vitro* transcribed and thus uniformly labelled RNA, we devised a novel assignment strategy that integrates intra- and inter-residual H1–H8 correlations based on a combination of NMR spectra to discern the Hoogsteen base pairing pattern of tetrads. This combination of well-established methods and newly adapted NMR pulse sequences enabled a straightforward and unambiguous assignment for non-site-specifically labelled G4s, a capability previously unavailable. Subsequent in-depth analysis and comparison with other triplanar RNA and DNA G4 structures reveal a unique feature of the core of the two *BCL2* G4 mutants and provide new insights into the role of the sugar conformation in G4s. This study not only enhances our understanding of the *BCL2* RNA G4 but also signifies a notable advancement in RNA G4 structural biology by facilitating NMR structure determination, a crucial step for obtaining more insights into these regulatory elements.

## Results and discussion

### Triple mutants trap the *BCL2* G4 in a single conformation

In 2010, a highly sequence- and site-conserved guanine-rich sequence was discovered in the 5′ UTR mRNA of the human B-cell lymphoma gene 2 (*BCL2*) (Fig. 1A).^20,58^ This gene encodes Bcl-2, an anti-apoptotic protein intricately regulated both transcriptionally and post-transcriptionally.^59^ Misregulation of this protein has been associated with neurodegenerative diseases such as Parkinson's and Alzheimer's,^60,61^ as well as with multiple lymphomas and resistance to anticancer therapies.^62,63^ Furthermore, the *BCL2* RNA G4 structure has been demonstrated to affect translation *in vitro*, making it a potential target for therapeutic intervention to regulate Bcl-2 protein levels.^20^ Indeed, three natural alkaloids (palmatine, nitidine, jatrorrhizine) with antiproliferative properties bind with high affinity to this G4,^64^ but their expensive synthesis poses a challenge for further research.^65^ Thus, a comprehensive understanding of the structural and dynamic features of this *BCL2* RNA G4 is essential to identify additional potential drug molecules and to develop alternative treatments targeting this regulatory RNA.

Initial analysis of the 25-nucleotide guanine-rich sequence revealed the formation of a stable parallel G4.^20^ However, the study did not disclose the highly dynamic nature of this sequence, which is capable of adopting multiple G4 structures. In general, proton resonances at 10–12 ppm indicate characteristic G4 formation and originate from the H1 protons of the guanines inside the core.^54^ The lack of distinct signals in this region for the initial wild-type sequence (25mer wt, in Fig. 1C) suggests various interchanging conformers dependent on the incorporation of different guanines inside the core. With such a dynamic behaviour, further analysis by NMR – let alone full structure elucidation – is not feasible. Therefore, we systematically restricted the intrinsic folding dynamics by reducing the number of excess guanines to obtain a single G4 conformation. An earlier study on a shortened wild-type sequence (22mer wt), in which two guanines at the 5′ end and one at the 3′ end were removed, also yielded a stable G4 structure.^58^ In contrast to the 25mer wt, the 22mer displays well-defined resonances in the respective region of the 1D ^1^H NMR spectrum (Fig. 1A and C), indicating a reduction in dynamics. The 22mer wt sequence consists of four segments with three to four consecutive guanines, likely forming a triplanar G4 and resulting in twelve H1 proton signals. The number of resonances indicates the presence of at least two conformations based on the incorporation of alternative guanines from within either the four-guanine stretch or the loop regions (Fig. 1A). To prevent guanine exchange between the loops and the G4 core, the guanines in the loops (G6 to A6 and G17 to U17) and the first guanine of the four-guanine stretch (G8 to A8 and U8, respectively) were mutated, yielding the mutants A6A8U17 and A6U8U17 (Fig. 1A). The ^1^H NMR spectra of the two triple mutant constructs indeed show well-resolved and sharp resonances corresponding to twelve H1, demonstrating that the G4 is trapped in a single triplanar conformation (Fig. 1C), an ideal case to establish a new assignment approach.

G4s tend to stack on top of each other (multimerisation), especially at high metal ion and nucleic acid concentrations. G-quadruplexes without flanking nucleotides (non-G nucleotides at 5′ or 3′ end) are even more prone to dimer formation.^66^ Here, RNA concentrations of ∼0.5–1.3 mM needed for high-resolution NMR spectra, together with a low K^+^ concentration of 2 mM, were applied. No indication of stable dimer formation upon varying RNA and salt concentration, or temperature (Fig. S2 and S3†) was observed. Additionally, the hydrodynamic radius of both mutants matches that of the monomeric 22mer wt (Fig. S4†).

CD spectra and melting curves confirm that at 2 mM KCl and pH 7, both mutants adopt parallel G4 structures with similar stability to the *BCL*2 G4 22mer and 25mer wild-type^20,58^ (*T*_m_ ∼ 60 °C, Fig. S1 and Table S1†).

### A universal assignment approach for G4 structures

The stacked tetrads are the central feature of all G4 structures, making them the ideal starting point for NMR assignment. While guanines in DNA G4s are typically unequivocally assigned by site-specific labelling, the assignment of guanines in RNA G4s relies primarily on intra- and interplanar H1–H8 NOE correlations. However, the limited signal dispersion poses a challenge, particularly in distinguishing between intra- and interplanar H1–H8 NOEs (see Fig. S10A and D†). To overcome this problem, we directly link the guanines within a plane *via* their H1 and H8 protons using *in vitro* transcribed uniformly labelled ^15^N-G or ^13^C,^15^N-G RNA. We combine established JR-HMBC and H8N7N2-COSY spectra with one of the two newly adapted pulse sequences, H1N1N2-COSY or H1(N1C2)N2, enabling unambiguous assignment of all guanines in the core and a direct connection to the H1–H8 NOESY region. By focusing solely on through-bond correlations, ambiguity is significantly reduced as only intraplanar H1–H8 correlations are revealed – a major advance in NMR structure determination of RNA G4s, particularly those with intricate structures or those exhibiting polymorphism.

**Fig. 2:**
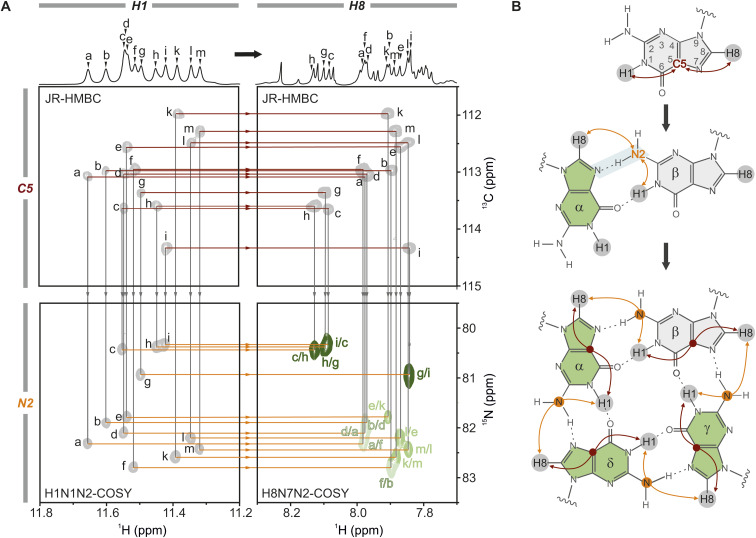
Unambiguous assignment of the tetrads. (A) The tetrad assignment is based solely on through-bond correlations, using three different spectra to establish both intra-residue (top panel, red lines) and inter-residue (bottom panel, orange lines) H1–H8 correlations. The intra-residue correlations are established *via* a JR-HMBC connecting the H1 (top left) and the H8 proton (top right) *via* the C5 carbon,^66–68^ and the inter-residue correlations *via* two HNN-COSY spectra. The H1N1N2-COSY (bottom left) connects H1 to N2 *via* a ^2H^J_NN_ scalar coupling (Fig. S4†), and the H8N7N2-COSY (bottom right) connects the H8 to N2 *via J*-coupling through the N7⋯H2–N2 hydrogen bond.^69^ The assignment is shown for A6A8U17 with the corresponding 1D ^1^H NMR spectra above, using a tentative proton assignment (a–m) for the twelve guanines of the core. Grey lines indicate the transfer of proton assignment to another spectrum. (B) Schematic representation of the consecutive steps required to establish the tetrad assignment. The protons circled in grey mark the detected protons, H1 and H8, respectively. The red lines mark the intra-residue correlations from H1 to H8 through C5, and the orange lines represent the inter-residue correlations from H1 to H8 through N2. The N7⋯H2–N2 hydrogen bond is marked in light blue. The JR-HMBC spectrum was recorded with 1.3 mM RNA, the HNN-COSYs using a 0.45 mM ^15^N-G-labelled RNA sample (H_2_O/D_2_O (9 : 1), 2 mM KCl, pH 7, 298 K).

**Fig. 3:**
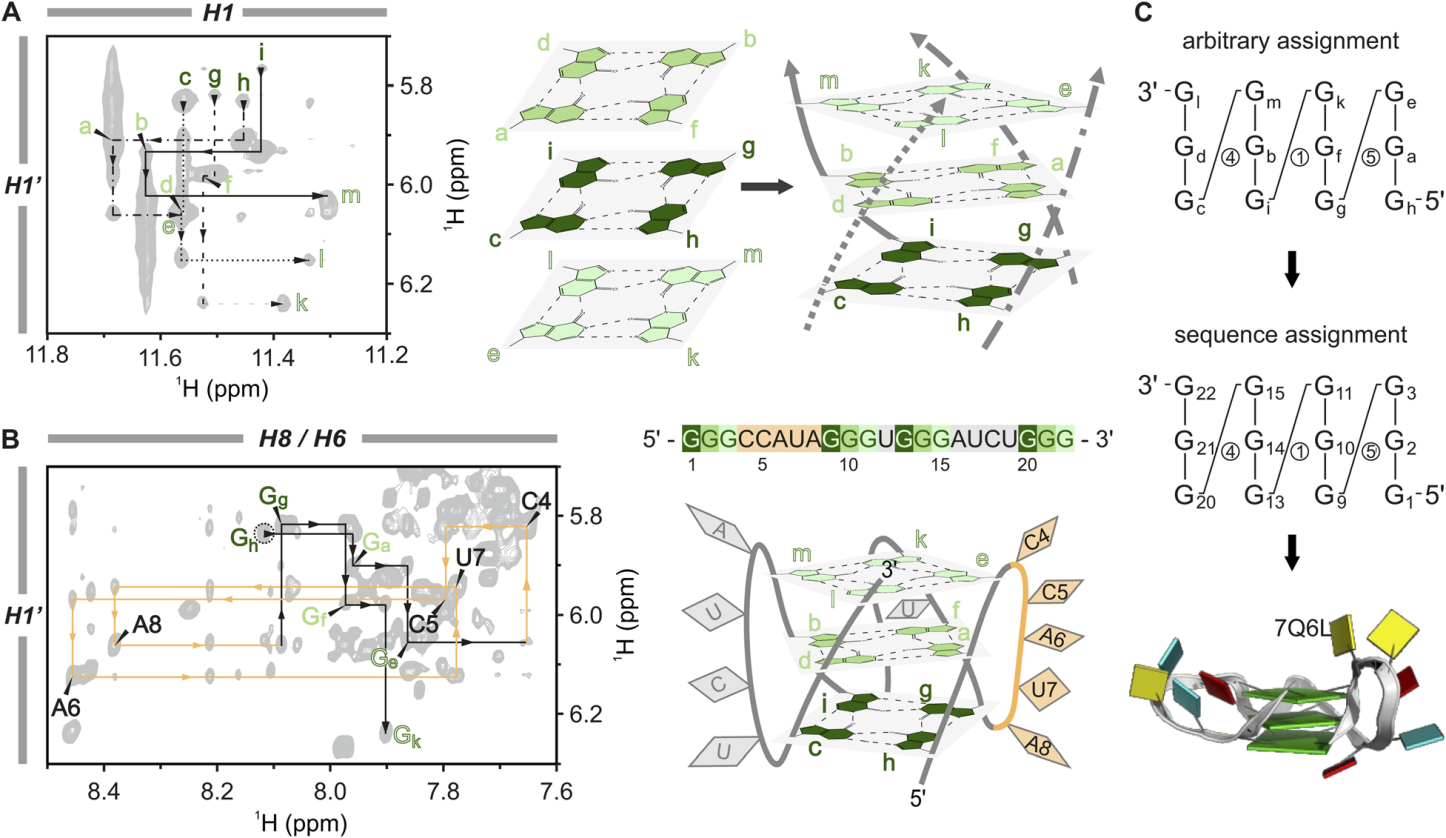
Sequential assignment and structural calculation of the A6A8U17 G-quadruplex construct. (A) H1–H1′ region of the [^1^H,^1^H]-NOESY (1.3 mM A6A8U17, H_2_O/D_2_O (9 : 1), 2 mM KCl, pH 7, 298 K) (left) used to assign the individual strands and subsequently to correctly order the tetrads in the core (right). Four line-types differentiate the individual strands, and tetrads are colour-coded with three shades of green. (B) Sequential walk region of the [^1^H,^1^H]-NOESY (1.3 mM A6A8U17, 100% D_2_O, 2 mM KCl, pD 7, 298 K). Showing the correlation of the sugar H1′ with the aromatic H8/H6 protons (left). The assignment of the loop nucleotides (CCAUA) is shown in orange, which was used to assign the strands in the correct order and to replace the tentative assignment of the guanines with the correct sequence numbering. The G4 sequence of the A6A8U17 mutant and a schematic structure with the tetrad guanines in three shades of green and the loop nucleotides in orange and grey, respectively (right). (C) A graphical shorthand notation introduced by Banco and Ferré-D’Amaré^89^ without sugar pucker information to show the transition from tentative to sequential assignment (circled numbers indicate loop lengths) (top) and block view^30^ of the calculated A6A8U17 *BCL2* mutant (PDB code: 7Q6L) (bottom).

Here, we use the A6A8U17 mutant as an example: we start with a tentative assignment of the H1 protons in the 1D ^1^H spectrum from left to right (guanines G_a_–G_m_). Using the JR-HMBC spectrum (Fig. 2A, red line in top spectra), the H1 protons are subsequently connected to the intra-residue H8 protons *via* C5 through-bond correlations (top Fig. 2B), allowing the tentative H1 assignment to be transferred to the H8 protons.^67–69^ In a second step, we employed an H8N7N2-COSY (Fig. 2A, grey line to the bottom right spectrum) to correlate the H8 protons to the N2 nitrogens of neighbouring guanines *via* the N7_(α)_⋯H21_(β)_–N2_(β)_ hydrogen bond (Fig. 2B).^70^ In principle, the O6_(α)_⋯H1_(β)_–N1_(β)_ hydrogen bond could also be used to link two guanines. However, the small coupling constant across this connectivity pathway results in insufficient sensitivity, rendering it unsuitable for acquiring high-quality NMR spectra.^54^ To complete the assignment of the H8–H1 inter-nucleotide linkage, we adapted two pulse sequences to detect the intramolecular H1–N2 correlations:^71,72^ H1N1N2-COSY (Fig. S5†) and H1(N1C2)N2 (Fig. S6†) (see also Fig. 2A bottom and Fig. 2B middle). High-resolution spectra were obtained with 0.4 mM ^15^N-G-labelled and ^13^C,^15^N-G-labelled RNA samples, respectively, within 1 hour measuring time. H1N1N2-COSY high-resolution spectra were also obtained at 323 K (Fig. S7†). Measuring at such high temperatures can substantially minimise the multimerisation effect, as G4 core stacking is reduced,^73,74^ which is particularly important when higher potassium(i) concentrations are required or when the G4 has a higher propensity for forming multimers. Using the H1N1N2-COSY (Fig. 2A, following the grey line to the bottom left spectrum), we established the intra-residual link between the H1–N2 and connected it to the inter-nucleotide N2–H8 correlation *via* the orange lines. The iterative process continues until all four nucleotides within each tetrad are assigned, as illustrated in detail in Fig. S8.† In the case of the A6A8U17 *BCL2* G4, the assignment of the three tetrads revealed the connections: G_a_–G_f_–G_b_–G_d_, G_c_–G_h_–G_g_–G_i_, and G_e_–G_k_–G_m_–G_l_ (Fig. 3A, left). The intraplanar H1–H8 (Fig. S10A†) and H1–H1 (Fig. S14†) through-space correlations in the NOESY spectrum confirmed this assignment.

In the next step, the individual tetrads are arranged to assemble the G4 core. Through H_2_O–D_2_O exchange experiments, we identified the middle tetrad (G_a_–G_f_–G_b_–G_d_) (Fig. S9†) characterised by slower deuterium exchange of the H1 protons due to reduced solvent exposure.^54^ The rest of the core was compiled using H1–H1′ NOE correlations, akin to the sequential walk (Fig. 3A, black lines in the spectrum). Unlike the crowded sequential walk regions H6/H8–H1′ and H6/H8–H2′ (Fig. 3B, S10C and S11†), which were used additively to verify the assignment, this NOESY region only displays guanine signals from the tetrads. Applying this approach, we established the order of the guanines along the four edges (5′–G_c_→G_d_→G_e_–3′, 5′–G_g_→G_f_→G_k_–3′, 5′–G_h_→G_a_→G_e_–3′ and 5′–G_i_→G_b_→G_m_–3′) and arranged the tetrads accordingly to form the core. The tetrad arrangement was further corroborated by interplanar H1–H8 NOEs (Fig. S10D†) and correlations between sugar H2′ to H1 of the stacking guanine from the left neighbouring strand indicated by dashed black lines in Fig. S10E and F.† This latter correlation illustrates the right-handed twist of the G4 core, a structural characteristic also typical for A- and B-helical nucleic acid structures.^75^

Finally, we replaced the tentative assignment with the correct sequence numbering by identifying the specificity from the connections between G-tracts and unique loop sequences. A6A8U17 contains three loops with different lengths and sequences (CCAUA, U, and AUCU, respectively; Fig. 3B). In principle, a single connection, *e.g.* a G to two consecutive cytosines (C4 and C5), is sufficient to convert the sequence assignment. In the present case, we could trace correlations over an entire loop with the sequential assignment of the first two G-tracts connected by the CCAUA loop (orange): 5′–G_h_–G_a_–G_e_–C4–C5–A6–U7–A8–G_g_–G_f_–G_k_–3′ (Fig. 3B). The base identities of the loop nucleotides were determined using [^13^C,^1^H]-HSQC experiments and then mapped to the NOESY sequential walk region (H6/H8–H1′ region), with the complete sequential assignment shown in Fig. S11.† The conversion from the tentative to the sequential assignment is depicted in Fig. 3C with G_h_ = G1, G_a_ = G2, G_e_ = G3, *etc.* The remaining proton resonances were assigned using additional 2D [^1^H,^1^H]-TOCSY and [^13^C,^1^H]-HMBC experiments. This comprehensive assignment process was performed for both mutants. Subsequently, the structural models of A6A8U17 (PDB ID 7Q6L)^76^ and A6U8U17 (PDB ID 7Q48)^77^ were calculated based on NOE-derived distance restrictions, dihedral angle restrictions, and the definition of hydrogen bonds in the G4 core (Table S2†).

### Analysis of the *BCL2* structures and comparison to other G4s reveals a particularly compact core

**Fig. 4:**
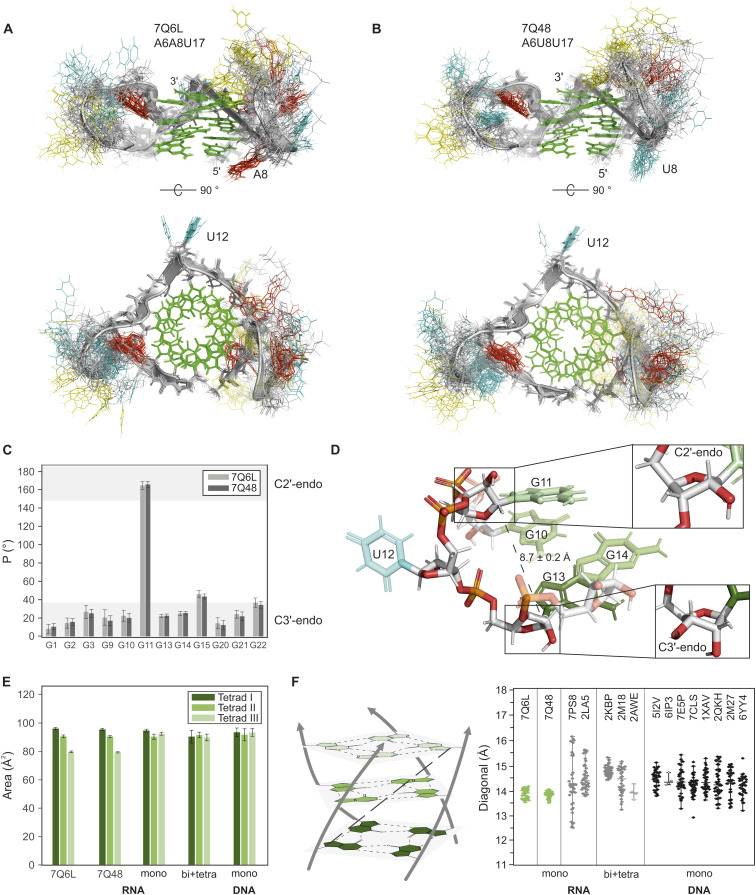
Structural analysis of the *BCL2* G-quadruplexes and comparison to other parallel G-quadruplex structures. (A and B) Top and side view of the overlay of the 20 lowest-energy structures of 7Q6L and 7Q48, respectively. Backbone and ribose sugars are depicted in grey, guanines in green, adenines in red, uracils in cyan, and cytosines in yellow. Some loop nucleotides are transparent for better visualisation of the core. (C) Histogram of the pseudorotation phase angle *P* of the twelve guanines in the core of the two mutants (7Q6L in grey; 7Q48 in dark grey). The preferred range of P for the endocyclic sugar conformations C3′-endo (0–36°) and C2′-endo (144–180°) is shown in light grey.^76^ (D) Close-up of the single-nucleotide loop U12 that bridges a C1′–C1′ distance of 8.7 ± 0.2 Å (dashed line) to connect the G9–G11 strand to the G13–G15 strand. The sugar pucker in G11 adopts a C2′-endo conformation (top inset), while G13 (bottom inset) and all other guanine sugar puckers adopt a C3′-endo conformation. Oxygen and phosphorus atoms are shown in red and orange, respectively, while the carbon atoms are grey. The colours of the bases are the same as given in A. (E) Tetrad areas of the two *BCL2* mutants 7Q6L and 7Q48 in comparison to the other monomolecular RNA G4s (7PS8 and 2LA5), the bi- and tetramolecular RNA G4s (2KBP, 2M18, 2AWE) and the monomeric, parallel DNA G4s. (F) Schematic representation of the core with the defined diagonal (dashed black line) (left) and a jitter plot (right) comparing the diagonals of the two *BCL2* mutants 7Q6L and 7Q48 (green) with the other mono-, bi- and tetramolecular RNA G4s (grey) and the monomeric parallel DNA G4s (black). Filled diamonds indicate NMR structures and unfilled diamonds represent crystal structures. The PDB codes of all structures are shown in the figure.

Superimposing the 20 lowest-energy models out of the 200 calculated for A6A8U17 and A6U8U17 reveals remarkably similar structures for both mutants (see Fig. 4A and B), characterised by well-defined G4 cores and flexible loops (RMSD: 3.29 ± 1.03 Å (A6A8U17) and 3.02 ± 0.68 Å (A6U8U17)). Notably, the rigid cores adopt highly similar structures, as evidenced by the low RMSD (0.57 ± 0.18 Å) upon superposition. The relatively high overall RMSDs are primarily attributed to the high structural diversity of the two longer loops, CCAUA(U) and AUCU. Nevertheless, several local structural features can be distinguished. The first loop CCAUA(U) connects G3 and G9, where the sequence difference at position 8 between the two mutants appears to exert only a local structural effect. In particular, A8 in A6A8U17 stacks on G1 and G9 from the 5′ tetrad (see Fig. 4A, top), which is well reflected by strong correlations between A8H2 and both G1H1 and G9H1 in the NOESY spectrum (red box in Fig. S10A†). In contrast, U8 in the A6A8U17 mutant lacks these long-range NOE correlations and instead displays only interactions between both neighbouring nucleotides (U7 and G9). This leads the U8 to prominently splay away from the G4 core (Fig. 4B, top), which is consistent with the weaker stacking interactions of pyrimidine rings compared to purines.

In the AUCU loop, connecting G15 and G20, a significant number of NOEs are detected in both mutants (A16 to U19), and A16 to C18 in the case of the A6U8U17 mutant. However, even with the inclusion of these long-distance NOEs, the structural diversity could not significantly be reduced. In contrast to the two longer loops, the single nucleotide loop U12 is predominantly stabilised in one orientation (Fig. 4A and B, bottom), pointed away from the G4 core, which is reflected in the NOESY data with only correlations of U12 with G11 and G13 present.

Analysis of the sugar conformation of the G4 core nucleotides *via* the pseudorotation phase angle *P* ^78^ shows that all guanines in both constructs adopt the typical C3′-endo sugar conformation, except for G11, which is exclusively in the C2′-endo conformation (Fig. 4C). Sugar puckers were restrained according to the TOCSY data (intensity of H1′–H2′/H3′, Fig. S13B†). In the case of G11, the TOCSY intensities were ambiguous. However, the HSQC showed a far upfield H1′–C1′ resonance characteristic for a C2′-endo sugar conformation^79^ (red box in Fig. S13A†). This C2′-endo sugar conformation is rather atypical for base-paired RNA regions, as it is destabilised by about 6 kcal mol^−1^ compared to the C3′-endo for steric and electronic reasons.^80^ Interestingly, G11 is immediately followed by the single-nucleotide loop U12, which spans a C1′–C1′ distance of 8.7 ± 0.2 Å to reach G13, the next guanine on the bottom tetrad (Fig. 4D). Generally, the C2′-endo conformation allows for a longer intra-strand P⋯P distance (C2′-endo: 7 Å; C3′-endo: 5.9 Å),^75,81^ which probably explains the C2′-endo sugar conformation of U12. The C2′-endo conformation of G11 also enables the downward orientation of its C3′ carbon toward G13 (Fig. 4D, top inset).

We expanded our analysis to include additional parameters for a comprehensive G4 core characterisation, encompassing the tetrad area and planarity. The tetrads were designated as tetrad I, II and III for the 5′-, the middle and the 3′-end tetrad, respectively. Calculation of tetrad areas based on N9 distances (Fig. S16A†) revealed an intriguing pattern for both *BCL2* constructs: tetrad I > tetrad II > tetrad III (Fig. 4E), with a decrease of approximately 10 Å^2^ between each tetrad. Since the hydrogen bond lengths remain consistent across all three tetrads, they cannot be responsible for the size difference. Overlaying the three tetrads indicates that the guanine bases in tetrad III are more tilted out of the plane than in tetrads I and II (Fig. S16C†). The planarity of the tetrads was assessed using the x3DNA-DSSR software,^30^ quantifying their deviation from perfect planarity. Despite employing the same low weighting in structural calculations, the planarity significantly differs between the planes (Table S3†). Specifically, the middle tetrad exhibits significantly higher planarity than the other two (planarity deviation: tetrad II < tetrad I < tetrad III). While this observation is anticipated, given its sandwich position, it fails to elucidate the specific trend observed for the tetrad area. Further analysis of backbone torsion angles and geometric parameters yielded no correlations, leaving the underlying reason for this behaviour unknown.

To assess if the observed trends are typical characteristics of G4s, we compared the two *BCL2* G4 constructs with other mono-, bi-, and tetramolecular RNA G4s, as well as parallel monomolecular DNA G4 structures. Among approximately 90 known RNA G4s, only a few are triplanar. We selected two bi- (2KBP, 2M18), one tetra- (2AWE), and two non-canonical monomolecular RNA G4s. One monomolecular RNA G4s (7PS8) is naturally occurring and similar in length, while a duplex stabilises the other (2LA5). Additionally, we chose seven triplanar DNA G4s with similar sequence lengths (5I2V, 6IP3, 7E5P, 1XAV, 2KQH, 2M27, and 6YY4), and one structure stabilised by a hairpin (7CLS).

Sugar pucker analysis revealed the expected pattern, with RNA G4s primarily adopting the C3′-endo conformation, whereas DNA G4s predominantly have the C2′-endo sugar pucker (Fig. S17†).^12,82^ Despite this tendency, our analysis disclosed numerous instances of the C2′-endo conformation in RNA G4s, especially near single-nucleotide loops and bulges. In addition, several NMR structures contain models with different sugar conformations for certain guanines as well as conformations other than the C2′-endo and C3′-endo sugar pucker. These observations show that the occurrence of C2′-endo conformations in RNA G4s is not exceptional and suggest that the adaptation of the sugar pucker is a general mechanism to accommodate specific structural features.

In the next step, we investigated whether the molecularity of the G4s or the preference for a sugar pucker influences the core structure. Analysis of the tetrad areas revealed no differences between RNA and DNA G4s, nor a tendency within the tetrads, suggesting that the observed decreasing trend in our structure was an exception rather than a general feature (Fig. 4E). We also determined the tetrad planarity and found that DNA G4s tend to have slightly more planar tetrads (Tables S3 and S4†). However, a direct comparison is not meaningful as X-ray structures generally show higher compactness and planarities. In addition, in NMR structure calculations, the planarities are often set manually.

The overall size of a G4 core mainly depends on the helical rise and the helical twist (Fig. S19†), with the diagonal serving as a key metric for this dimension. This diagonal corresponds to the distance between the N9 atoms of a guanine in the 5′ tetrad and a guanine on the right-hand neighbouring strand of the 3′ tetrad and is the largest distance in the G4 core (Fig. 4F, left). The four diagonals of each structure were analysed to evaluate the core size. This approach has the advantage that the diagonal, in contrast to the helical rise and the helical twist, is a measure that can also be used to compare G4s with bulges, different strand directions or other non-canonical features. While a general comparison suggests no significant differences between mono-, bi- and tetramolecular RNA G4s, the two *BCL2* structures with minimal diagonal lengths and a narrow distribution (A6A8U17: 13.9 ± 0.4 Å; A6U8U17: 13.8 ± 0.3 Å) stand out as notably compact and symmetrical. The two other monomolecular RNA G4s, 7PS8 and 2LA5, exhibit a less compact core and a distinctively larger distribution of diagonal lengths, most likely due to their non-canonical G4 nature. Unlike canonical G4s, having only consecutive guanines and short loops (<7 nucleotides),^83^ non-canonical structures contain additional structural features such as interruptions by bulges,^84^ longer loops or elongated 5′ and 3′ ends that can promote additional secondary structures.^85^ Among the RNA G4s, 7PS8 is most similar to the *BCL2* structures (Fig. S17–S19†). However, the presence of a three-nucleotide bulge in the second G-strand seems to push the guanine coming after the loop slightly out of the tetrad, affecting the corresponding diagonal. Comparing the two *BCL2* structures with canonical monomolecular parallel DNA G4s underlines the exceptional compactness and symmetry of their cores. In DNA, G4s greater variability in sugar conformation may account for the larger core and the generally wide distribution of diagonal lengths.

## Conclusions

Acute lymphoblastic leukaemia (ALL) is the most prevalent form of cancer and leukaemia in children. The remarkable efficacy of Bcl-2 inhibitors in ALL treatments underscores the importance of reducing Bcl-2 protein levels to facilitate apoptosis. Currently, treatments are limited to agents that exclusively act at the protein level.^86–88^ However, the regulatory involvement of the 5′ UTR *BCL2* mRNA G4 in Bcl-2 protein expression presents a promising alternative target for future drug development. A crucial first step in this direction is to understand the structural and dynamic properties of these small regulatory elements, which is best achieved by NMR spectroscopy. However, the highly dynamic nature of the *BCL2* RNA G4 (25mer wt) leads to extensive resonance overlap and signal broadening. By truncating and mutating the sequence, we have restricted the G4 to a single conformation and solved one of the first monomolecular RNA G4 structures by applying a new approach for the unambiguous assignment of the core guanines. This method requires no site-specific labelling but utilises uniformly isotope-labelled RNA, along with three aligned NMR spectra and is not only applicable for any G4 type but also holds promise for other Hoogsteen hydrogen bonding motifs, such as A(GGGG)A hexads, CGG or CGA^+^ triplexes.

Although structural diversity has been dramatically expanded in recent years by non-canonical motifs,^36,89^ structural comparison with other published parallel triplanar G4s revealed no recognisable structural features of the core specific to RNA or DNA G4s or distinguishing mono- from bi- or tetramolecular structures. Despite RNA G4s favouring C3′-endo sugar puckers and DNA G4s C2′-endo sugar puckers, the sugar pucker is less rigidly defined compared to canonical base pairing, where it dictates the overall structure of the double helix as A- or B-type. Instead, sugar pucker adaptation allows the inclusion of restrictive structural features such as single nucleotide loops and bulges.

The coexistence of multiple G4 structures of the wild-type *BCL2* RNA G4 sequence *in vitro*, together with the highly dynamic loop structures observed in the two triple mutants, suggests a complex scenario *in vivo*. While this complexity poses a challenge, it also presents intriguing opportunities for drug development as the intrinsic folding dynamics of G4 structures may influence their function by creating distinct molecular recognition sites for natural or artificial biomolecules, including binding proteins, miRNAs, or drug molecules. Therefore, further structural studies of the *BCL2* RNA G4 should focus on the elucidation of the different conformers of the wild-type sequence and their dynamic relationship. This novel assignment strategy, allowing for unambiguous assignment of the core guanines, has the potential to elucidate such complex RNA G4 systems in the future, thereby advancing insights into their intrinsic dynamics. This knowledge is crucial for a better understanding of their biological function and a first step in the process of producing new targeted cancer treatments including G4s.

## Author contributions

Z. W. and C. F. R. contributed equally to this work. Z. W., C. F. R., A. D. M., S. Jo., and R. K. O. S. conceived the project; A. D. M. developed, isolated, and measured the first NMR constructs; Z. W. and S. Ju. developed the methodology; Z. W. and C. F. R. performed the formal analysis, investigation, and validation of the data with assistance from S. Ju. and S. Jo.; S. Jo. and R. K. O. S. administered and supervised the project and helped to interpret the results; C. F. R. and S. Jo. wrote the original manuscript; all authors reviewed and edited the manuscript.

## Conflicts of interest

There are no conflicts to declare.

## Supplementary Material

SC-016-D5SC01416F-s001

## Data Availability

Structure coordinates, NMR chemical shifts and spectral peak lists for the *BCL-2* RNA A6A8U17 and A6U8U17 mutants have been deposited in the RCSB Protein Data Bank under the PDB IDs 7Q6L and 7Q48.^76,77^ NMR chemical shifts and spectral peak lists are also available from the Biological Magnetic Resonance Data Bank (BMRB) under the IDs 34676 and 34674. Other data supporting this article have been included as part of the ESI.†
