# Supplementary material for: Elucidating the solution structure of the monomolecular BCL2 RNA G-quadruplex: a new robust NMR assignment approach
Source: Chem Sci. 2025 Mar 26;16(22):9669–78. doi: 10.1039/d5sc01416f (PMC11962745; doi:10.1039/d5sc01416f)
Supplement: SC-016-D5SC01416F-s001 [file SC-016-D5SC01416F-s001.pdf]

Supplementary Information (SI) for Chemical Science.
This journal is © The Royal Society of Chemistry 2025

Supplementary Information for

Elucidating the Solution Structure of the Monomolecular *BCL2* RNA G-quadruplex: A New Robust NMR Assignment Approach

Author list

Zenghui Wang[1]†, Carla Ferreira Rodrigues[1]†, Simon Jurt[1], Alicia Dominguez-Martin[2], Silke Johannsen[1]*, Roland K.O. Sigel[1]*

Affiliations

[1]Department of Chemistry, University of Zurich; 8057 Zürich, Switzerland.

[2] Department of Inorganic Chemistry, Faculty of Pharmacy, University of Granada; 18071 Granada, Spain.

*Corresponding authors. Email: silke.johannsen@chem.uzh.ch, roland.sigel@chem.uzh.ch

†These authors contributed equally to this work.

The file includes:

Experimental Section

Supplementary Figs. S1 to S19

Supplementary Tables S1 to S4

Table of Content

Experimental section 3

Sample preparation 3

UV melting studies 3

Circular Dichroism (CD) 4

NMR measurements 4

Structural calculation 5

Structural analysis 6

Additional Data 7

CD Measurements 7

Confirmation of monomeric structure 8

H1N1N2-COSY pulse sequence 11

H1(N1C2)N2 pulse sequence 13

Spectral quality and assignments 15

Methodology for G-quadruplex tetrad assembly 16

Exchange experiments 17

Tetrads assembly and sequential assignment of the G4 core by NOE correlations 18

Sequential Assignment 19

Glycosidic bond orientation 20

Sugar conformation 21

NOE assignment of the G-quadruplex core 22

Calculated BCL2 structures 23

Area and planarity of the BCL2 G-quadruplex tetrads 24

Schematic structures of all analysed G-quadruplexes 25

Overlay of all NMR calculated G-quadruplex structures 26

Comparison of geometrical G4 core parameters 27

NMR restraints and structural statistics 28

Planarity 29

References 30

Experimental section

Sample preparation

All chemicals were at least purists. p.a. and purchased from Fluka – Sigma-Aldrich (Buchs, Switzerland) or Brunschwig Chemie (Basel, Switzerland). DNA templates were obtained PAGE-purified from Microsynth (Balgach, Switzerland) with the top strand (ts) containing only the complementary promoter sequence and the first two Gs, and the template strand (ot) having 2'-methoxy modifications at the two terminal nucleotides to prevent 3'-heterogeneity of the RNA.[1] Nucleoside 5'-triphosphates (NTPs) at natural isotope abundance (NA) were purchased from Roth AG (Arlesheim, Switzerland), and ^{13}C-, ^{15}N-, or ^{13}C,^{15}N-labelled NTPs (isotopic enrichment of all NTPs: > 98 atom%) were obtained from Silantes GmbH (München, Germany). All RNA samples were transcribed *in vitro* with homemade T7 RNA polymerase using natural abundance or labelled NTPs and purified as previously described.[2] A 10 mL *in vitro* transcription yields, on average, about 150-200 nmol RNA, independent of the type of NTP used. Transcription mixtures generally contained 10 – 20 mM GTP and 5 mM of the other three NTPs, 0.3 μM of the DNA templates, 0.01% Triton-X, 50 mM $MgCl_2$, 200 mM Tris-HCl pH 7.5, 200 mM DTT, 10 mM Spermidine, and a suitable amount of the T7 RNA (the amount was adapted according to the activity of each enzyme batch). Some transcriptions were performed including in addition 5 mM GMP, resulting in RNA with a 5'-monophosphate terminus. All transcripts were purified by 20% denaturing polyacrylamide gel electrophoresis (PAGE), recovered from the gel by electroelution (Elutrap Electroelution System, Whatman, UK), desalted with Vivaspin® concentrators (Sartorius Stedim Biotech S.A., Stonehouse, UK) with a molecular weight cut-off of 3 kDa by washing twice with 1 M KCl pH 8 and 6 – 8 times with ddH_2O, lyophilised, and resuspended. The correct mass of the final isolated RNAs was verified by a Bruker Autoflex Speed MALDI-TOF mass spectrometer (Bruker, Massachusetts, USA) in the negative-ion mode (A6A8U17 (7Q6L)[3]: calc. 7.45 kDa; meas. 7448 m z^{-1} and A6U8U17 (7Q48)[4]: calc. 7.43 kDa; meas. 7423 m z^{-1}). The RNA concentrations were determined by measuring the absorbance at 260 nm using the extinction coefficients (ε_{260}): 220.4 mM^{-1} cm^{-1} for A6A8U17 (7Q6L) and 215.9 mM^{-1} cm^{-1} for A6U8U17 (7Q48), which were calculated with the *RNA Molecular Weight Calculator* (AAT Bioquest, https://www.aatbio.com/tools/calculate-RNA-molecular-weight-mw). Before measurements, the RNA G4s were annealed by heating at 95 °C for 3 min and then slowly cooling to room temperature.

UV melting studies

Temperature-dependent absorbance measurements were performed on a Cary 3500 UV-Vis spectrophotometer (Varian Inc., Palo Alto, USA) using a 10 mm quartz cuvette with a total volume of 1 mL. The samples contained 4 μM RNA G4 in 2 mM KCl at pH 7, were degassed for 30 s, and covered with paraffin oil before measurement. Absorbance at 295 nm was recorded every 0.1 °C during two heating and two cooling cycles from 25 °C to 90 °C or 95 °C and from 90 °C or 95 °C to 25 °C using a rate of 0.5 °C min^{-1}. *Melting temperatures* T_m were determined using the baseline method previously

described.[5] The results of at least two independent measurements were averaged, and the stated errors correspond to 2σ.

Circular Dichroism (CD)

CD spectra were recorded with a Jasco J-810 spectropolarimeter (Jasco Inc., Easton, MD) using a 10 mm quartz cuvette with a total volume of 1 mL. Each sample contained 4 μM RNA G4 in 2 mM KCl at pH 7. The CD measurements were performed at 25 °C in the 200 – 320 nm range at a scan rate of 50 nm min^{-1}, data pitch of 0.5 nm, bandwidth of 10 nm, and response time of 1 s. The spectra were baseline-corrected to a blank of 2 mM KCl at pH 7 and averaged from three individual continuous scans.

Temperature-dependent CD measurements were performed in the range of 20 °C to 95 °C using a heating rate of 10 °C h^{-1} and recording the CD signal at 263 nm every 0.2 °C. The melting temperatures T_m were determined as described for the UV melting studies. The results of two independent measurements were averaged, and the stated errors correspond to 2σ.

NMR measurements

NMR samples were prepared by dissolving the RNA in 220 μl H_2O/D_2O (9:1) or D_2O containing 2 mM KCl at pH 7. All NMR measurements were performed either on a Bruker AV-600 MHz equipped with a TCI z-axis pulsed field gradient CryoProbe® or on a Bruker AV-700 MHz spectrometer equipped with a TXI z-axis pulsed field gradient CryoProbe®.

The bases in the core were identified using intra- and interresidue H1 – H8 correlations. Intraresidue H1 – H8 correlations were determined using a JR-HMBC by linking the H8 – C5 correlations to the H1 – C5 correlations within each guanine.[6] The base pairing pattern within the tetrads was established on ^{15}N-G-labelled RNA by direct detection of N7···H21–N2 hydrogen bonding between every two adjacent guanines using two complementary pulse sequences. A H8N7N2-COSY was used to correlate H8 to the N2 of the neighbouring guanine[7] and H1N1N2-COSY for ^{15}N-G labelled RNA samples or H1(N1C2)N2 experiment for ^{13}C,^{15}N-G labelled RNA samples were applied to establish the intraresidue H1 – N2 correlation (for details, see Fig. S2 and S3).

A 2D [^{1}H,^{1}H]-NOESY spectra (150 ms, 350 ms mixing time) with a 1-1 spin-echo pulse sequence for water suppression in H_2O/D_2O (9:1) at 298 K was used to assign the exchangeable proton resonances. Non-exchangeable proton resonances were assigned from 2D [^{1}H,^{1}H]-NOESY (250 ms and 350 ms mixing time) with gradient excitation sculpting water suppression, 2D [^{1}H,^{1}H]-TOCSY (50 ms mixing time), [^{13}C,^{1}H]-HSQC (145 Hz coupling constant), and [^{13}C,^{1}H]-HMBC[8] spectra in D_2O at 298 K. Additionally, nucleotide-specific ^{13}C or ^{13}C,^{15}N labelled RNA samples were used for constant-time [^{13}C,^{1}H]-HSQC (155 Hz coupling constant) spectra to facilitate the spectral assignment.[9,10]

^1H chemical shifts were directly referred to external DSS (0.2%, pH 7.5), and ^{13}C and ^{15}N were indirectly referred to the ^1H resonance of DSS.[11] All NMR data was processed with Topspin 4.0.6 (Bruker, Massachusetts, USA) and analysed with NMRFAM-Sparky.[12]

Structural calculation

NOE distances were estimated from the 2D [^1H,^1H]-NOESY spectra in D_2O (250 ms mixing time) or H_2O/D_2O (9:1) (only with 150 ms mixing time). Integrated peak volumes were obtained in Sparky, and the distances were calibrated with the CALIBA macro from DYANA (ETH Zurich)[13] using the H5–H6 distance of pyrimidine bases as a reference (2.47 Å). NOE correlations of non-exchangeable protons were grouped according to their strength, corresponding to strong (1.8 – 3.0 Å), medium (1.8 – 4.5 Å), weak (3 – 6 Å), and very weak (4 – 7 Å). Exchangeable protons were categorised into strong (2.3 – 4.8 Å), medium (3.0 − 5.8 Å), and weak (4.0 − 7.2 Å) correlations. Distances between atoms involved in the *Hoogsteen* hydrogen bonds of the tetrad were restrained to ideal hydrogen bond lengths, using two RNA G4 solution structures (PDB ID: 2KBP and 2M18) as reference. As usual, the triphosphate at the 5' end of the sequence was omitted, as no structural information is obtained from NMR.

The glycosidic torsion angles were restrained according to the intensity of the intraresidue H1' – H6/H8 NOEs and the H1' – C6/C8 and C1' – C2/C4 correlation in the [^{13}C,^1H]-HMBC spectra.[8] Generally, very strong H1' – H6/H8 correlations in the NOESY and the presence of C1' – C2/C4 correlations in the HMBC indicate *syn* conformation. No strong signals were visible in the NOESY spectra; however, weak C1' – C2/C4 correlations were observed for some nucleotides. Those nucleotides were left unrestrained; all others were loosely set to anti-conformation. A6U8U17 (7Q48): C5, A6, U7, U8, U12, A16, U17, and U19 were left unrestrained, all guanines (G1, G2, G3, G9, G10, G11, G13, G14, G15, G20, G21, G22), C4 and C18 were set to anti (χ = 240 ± 70 °). A6A8U17 (7Q6L): C5, A6, U7, U12, A16, U17, and U19 were left unrestrained, all guanines (G1, G2, G3, G9, G10, G11, G13, G14, G15, G20, G21, G22), C4, A8, and C18 were set to anti (χ = 240 ± 70 °).

The sugar puckers were restrained based on the intensity of H1' – H2' and H1' – H3' correlations in 50ms-mixing time 2D [^1H,^1H]-TOCSY spectra: strong H1' – H2' and H1' – H3' correlations indicate a C2'-endo sugar puckering (δ = 145 ± 15 °, ν_1 = 25 ± 15 °, ν_2 = – 35 ± 15 °), as was the case for C4, C5, U7, U/A8, U12, U17, C18, and U19. Intermediate H1'-H2' correlations such as G11 and A16 in A6A8U17 (7Q6L) were left unrestrained, and all other residues were restrained to C3'-endo (δ = 85 ± 15 °, ν_1 = – 25 ± 15 °, ν_2 = 37.3 ± 15.0 °). For the residues 6A, 8A, and G22 in A6A8U17 (7Q6L) and G22 in A6U8U17 (7Q48), the deviation range for the sugar pucker (angles δ, ν_1 and ν_2) was set to ± 25 °. The backbone angles α, β, γ, ε, and ζ were left unrestrained for all nucleotides.

Structure calculations were performed with Xplor-NIH 3.0 (National Institute of Health) using standard implemented force field parameters.[14–16] 200 preliminary structures were calculated via

simulated annealing, where the coordinates were randomised before elevating the temperature to 3500 K. During the simulated annealing the temperature was reduced from 3500 K to 25 K before a final torsion angle energy minimisation was performed. The experimental restraints for the calculation were enforced by the following force constants: NOE and hydrogen bonding distance restraints (ramped from 2 to 30 kcal/mol/$Å^2$), planarity restraints (2 kcal/mol/$Å^2$ for each quartet), and dihedral angle restraints to define base conformation and sugar puckers (200 kcal/mol/rad^2). The 20 lowest-energy structures were used for further structural refinement using the RNA-ff1 force field, designed explicitly for RNA structure calculation.[17] 200 refined structures were calculated, and the 20 best-refined structures (with the lowest energies and no distance and dihedral angle restraint violations) were visualised by Pymol[18] and further analysed.

Structural analysis

Structural analysis was performed on the two *BCL*2 G4s A6A8U17 (7Q6L) and A6U8U17 (7Q48) and other tri-planar RNA G4s as well as tri-planar, parallel-stranded DNA G4s. The G4s chosen for comparison were taken from the G4DB.[19] The PDB codes for the RNA G4s are: 2M18 and 2KBP (bimolecular, NMR), 2AWE (tetramolecular, X-ray diffraction), 2LA5 (monomolecular, antiparallel, G4-duplex, NMR), and 7PS8 (monomolecular, NMR); the DNA G4 PDB codes are 1XAV, 2KQH, 2M27, 5I2V, 6YY4, 7E5P (monomolecular, NMR), 7CLS (monomolecular G4-duplex, NMR), and 6IP3 (monomolecular, X-ray diffraction). x3DNA-DSSR[20] was used for the extraction of all conceivable G4 geometric parameters (planarity, helical twist, helical rise, shift, slide, rise, tilt, roll, twist, inclination, tip, backbone torsion angles (α, β, γ, ε, ζ, χ), endocyclic sugar torsion angles (ν_0, ν_1, ν_2, ν_3, ν_4), the phase angle of pseudorotation, and the puckering amplitude). In general, only the cores were used for comparison, as the flexibility of the loops makes it challenging to compare the structures. The geometric parameters of the NMR structures were averaged, and the standard deviation was calculated. Due to flexibility in the backbone torsion angles, averaging is not possible. The angles were clustered into specific regions and then compared.

Further determination of distances, such as the calculation of diagonals and tetrad areas, was achieved with Matlab[21] (plane corners were set to the C8 atoms) by using the coordinates from the PDB files of the calculated structures.

Block view visualisation of the solved G4 was done with the DSSR-PyMOL integration of Lu.[19]

Additional Data

CD Measurements

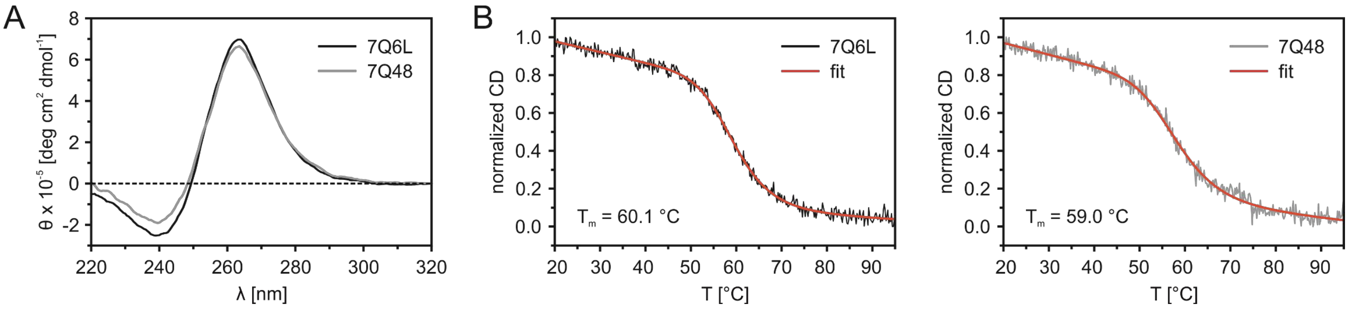

Figure S1. **CD spectra and thermal melting of A6A8U17 and A6U8U17.** A) Representative CD spectra of A6A8U17 (dark grey, 7Q6L) and A6U8U17 (light grey, 7Q48) in 2 mM KCl, pH = 7 show the characteristic parallel G4 pattern with a negative peak at 240 nm and a positive peak at 265 nm. B) Representative CD thermal melting curve of A6A8U17 (left) and A6A8U17 (right).

Table S1: Stability of A6A8U17 and A6U8U17 determined by thermal melting using CD and UV spectroscopy. Average melting temperatures (T_m) are the mean of at least two individual measurements, and the errors correspond to 2σ. In addition, T_m values of the *BCL2* 25mer and 22mer wild-type sequences are given for comparison.

***BCL2* G4 constructs**	**A6A8U17**	**A6U8U17**	**22mer wt**		**22mer wt[22]**	**25mer wt[23]**
KCl in mM	2	2	2	5	5	1
CD, T_m in °C	60.7 ± 1.8	58.9 ± 1.2	-	-	-	-
UV, T_m in °C	61.7 ± 1.5	59.5 ± 2.6	59.5 ± 2.8	62.8 ± 2.6	~ 57	59 ± 1

Confirmation of monomeric structure

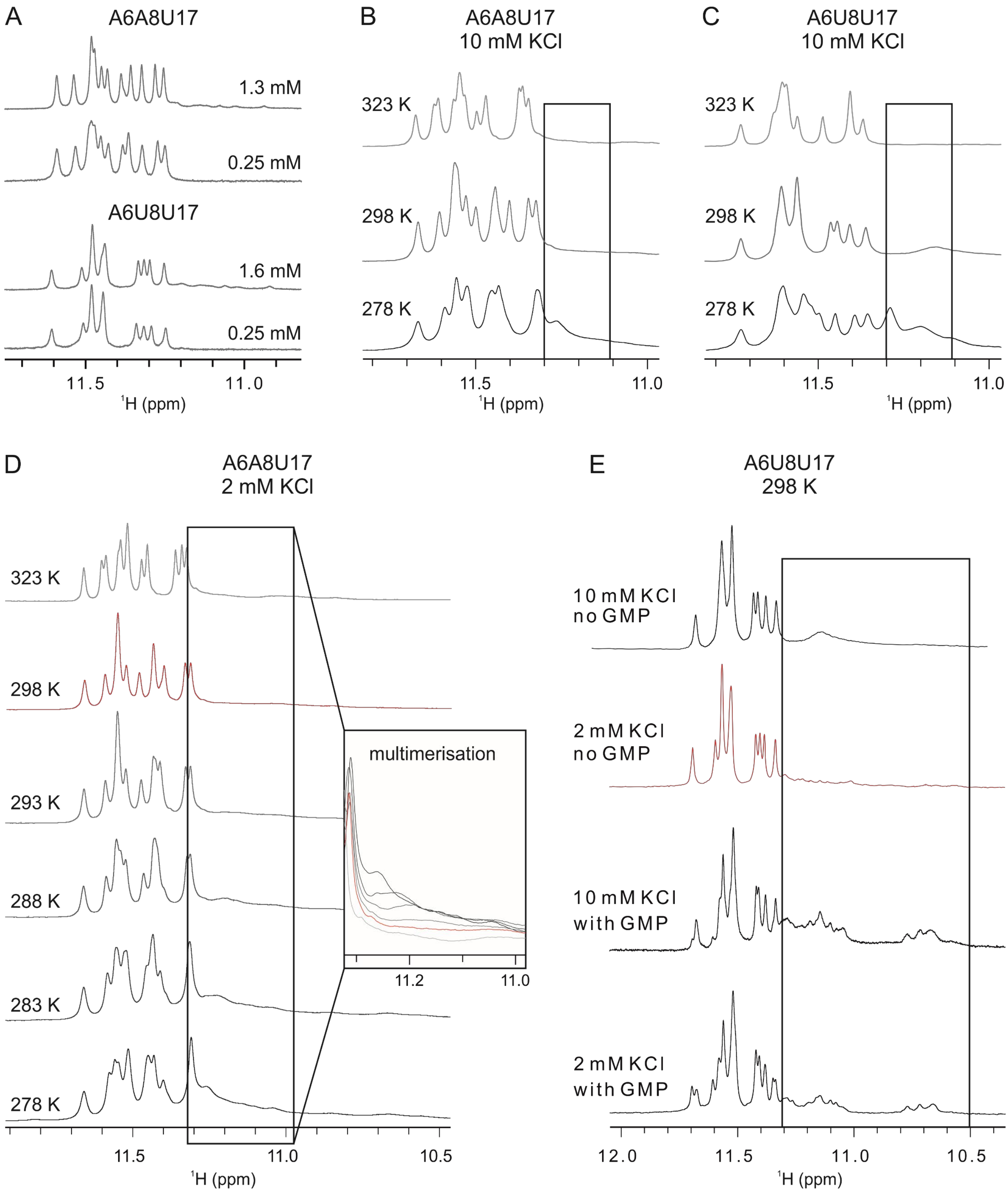

Figure S2. ^{1}H NMR analysis of A6A8U17 and A6U8U17: effects of RNA concentration, salt concentration, and temperature on monomeric stability A) Imino region of the ^{1}H NMR spectra of A6A8U17 and A6U8U17 at low (0.25 mM) and high (1.3 mM and 1.6 mM, respectively) RNA concentrations in H_2O/D_2O (9:1) with 2 mM KCl, pH 7. $^{13}C,^{15}N$-decoupled spectra at low RNA concentration were obtained with a $^{13}C,^{15}N$-A-labelled (A6A8U17) and a $^{13}C,^{15}N$-G-labelled (A6U8U17) sample, respectively. High RNA concentration spectra were acquired with samples at natural isotope

abundance. Small multimerisation resonances were observed at around 11 ppm in the high-concentration spectra, however, no significant chemical shift changes were observed. B+C) Imino region of the ^{1}H NMR spectra of A6A8U17 (0.5 mM) and A6U8U17 (0.3 mM) in 10 mM KCl at different temperatures. Boxed areas highlight the stacking interactions, which are enhanced at higher KCl concentrations but diminish with increasing temperature. D) Imino region of the ^{1}H NMR spectra for A6A8U17 (0.5 mM) across a temperature range of 278–323 K. The spectrum at room temperature (highlighted in red) serves as the reference for all subsequent experiments. Spectra at higher temperatures are shown in progressively lighter shades of grey. The inset focuses on the 11.0–11.3 ppm region, showing the gradual loss of stacking interactions with increasing temperature. E) Comparison of the imino region of A6U8U17 RNA transcribed without (top two spectra) and with 5 mM GMP (bottom two spectra; see Experimental Section for preparation details). RNA transcribed with GMP exhibits significantly enhanced stacking interactions, likely due to the predominant presence of a monophosphate group at the 5′-end. In contrast, the higher-charged triphosphate appears to prevent the exertion of stacking effects involving the 5′-end tetrads. The spectrum in red represents the standard conditions used for all subsequent experiments. All spectra were acquired in H_2O/D_2O (9:1) at pH 7.

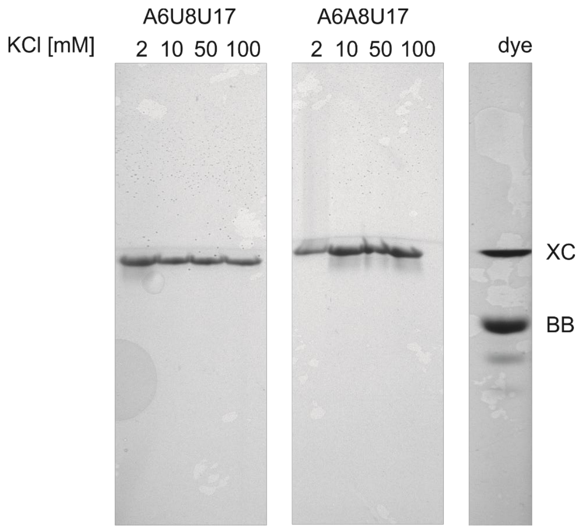

Figure S3. Native gel analysis of A6A8U17 and A6U8U17 at different KCl concentrations. 50 µM A6A8U17 (left) and 30 µM A6A8U17 (right) at four different KCl concentrations (2 mM, 10 mM, 50 mM, and 100 mM). Only a single band is visible, even at higher KCl concentrations, indicating that there is no monomer-dimer equilibrium under these conditions. XC: xylene cyanol; BB: bromophenol blue.

A

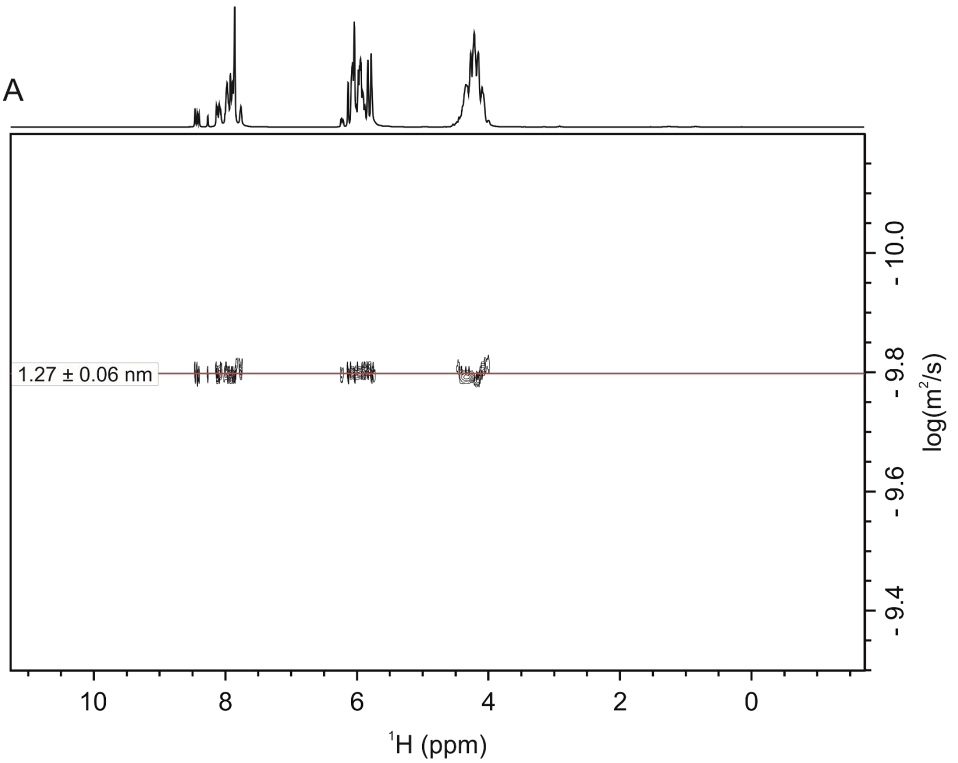

B

construct	r_H (nm)
22mer wt	1.31 ± 0.09
A6A8U17	1.27 ± 0.06
A6U8U17	1.37 ± 0.16

Figure S4. Hydrodynamic radii of A6A8U17 and A6U8U17 determined by DOSY. A) DOSY spectrum of A6A8U17 (0.5 mM (NA) RNA, 100% D_2O, 2 mM KCl, pD 7, 298 K. The red line indicates the only population detected during the measurement. B) Table of the hydrodynamic radii of the 22mer wt, A6A8U17, and A6U8U17 RNA G-quadruplexes. The radii were calculated from seven peaks in the range of 7.7-8.5 ppm using the Stokes-Einstein equation. The errors given correspond to three standard deviations (3σ). The hydrodynamic radii of all three constructs are the same within the error limits and align well with the estimated core diagonal of 1.39 ± 0.04 nm for monomeric A6A8U17 and 1.38 ± 0.03 nm for A6U8U17 (Figure 4).

H1N1N2-COSY pulse sequence

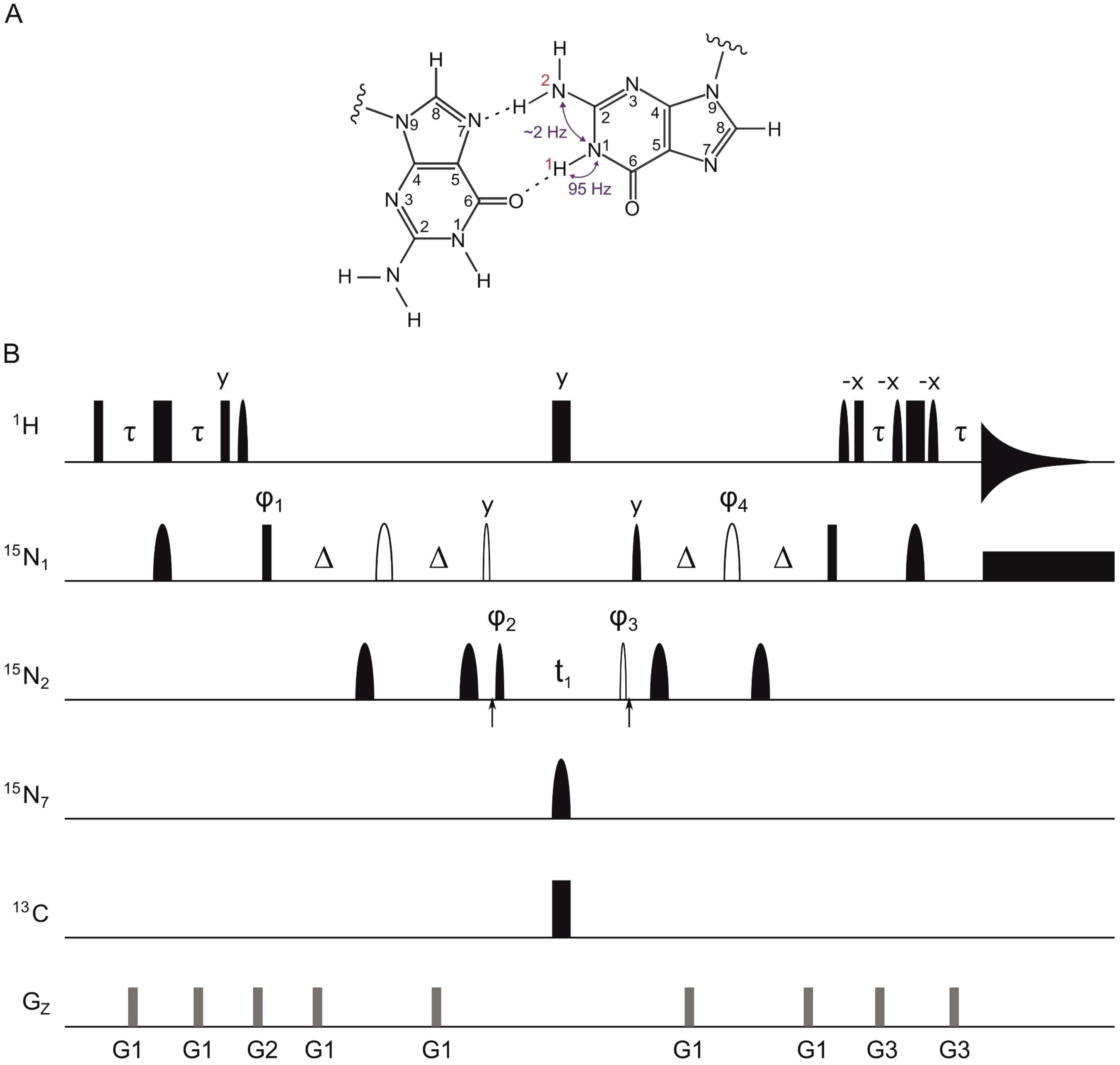

Figure S5. H1N1N2-COSY pulse sequence and important coupling constants. (A) The schematic illustration of the magnetisation transfer pathway for the H1N1N2-COSY with a $^{1}J_{HN}$ coupling of 95 Hz and a $^{2}J_{NN}$ coupling of ~2 Hz.[24] (B) The NMR pulse sequence of the H1N1N2-COSY correlating H1 and N2 of guanine. Narrow (wide) squares and sine-lobes represent 90° (180°) rf-pulses applied along the x-axis unless indicated otherwise. ^{1}H, ^{13}C, and ^{15}N hard pulses are applied at maximum rf power of 35.7 kHz, 16.7 kHz, and 6.8 kHz, respectively. The ^{1}H and ^{13}C carriers are placed at 4.7 ppm (water) and 85 ppm, respectively. For the N1-N2 coherence transfer, a pseudo-heteronuclear conversion is achieved by switching the position of the ^{15}N carrier from 145 ppm to 80 ppm before the 90° pulse of ϕ_2 and back to 145 ppm after the 90° pulse of $\phi_{3,}$ as indicated by the arrows. The shaped ^{1}H pulses are 1 ms sinc-pulses applied to the water resonance in order to maintain the water magnetisation along the z-axis during most of the experiment.[25] Filled and unfilled 180° ^{15}N shaped pulses are 0.9 ms I-BURP-2[26] and 0.62 ms r-SNOB[27] pulses, while filled and unfilled 90° ^{15}N shaped pulses are 1.2 ms gaussian cascade Q5[28] and time-reversed[29] Q5 pulses, respectively. 4.5 kHz and 1.1 kHz GARP decoupling[30] is applied on ^{13}C and

^{15}N nuclei during acquisition, respectively. The delays are: τ = 2.7 ms, Δ = 80 ms. The phase cycles are: ϕ_1 = x,-x, ϕ_2 = 2(x),2(-x), ϕ_3 = 4(x),4(-x), ϕ_4 = 8(x),8(y), ϕ_{rec} = ABBA with A = x,2(-x),x and B = -x,2(x),-x. Quadrature detection in F1 is achieved by States-TPPI of ϕ_2.[31] All pulsed field gradients have a duration of 1 ms, are of 10 % smoothed square-sine shape, and their strengths are G1 = 20 G/cm, G2 = 40 G/cm, G3 = 8 G/cm. For single labelled ^{15}N samples at ^{13}C natural abundance, the pulse on the carbon channel can be omitted. In the case of a more severe solvent exchange of the imino proton, it might be beneficial to refocus H1N1 antiphase magnetisation after the initial INEPT step, applying ^1H decoupling, and to re-create H1N1 antiphase magnetisation just before the back-INEPT.

H1(N1C2)N2 pulse sequence

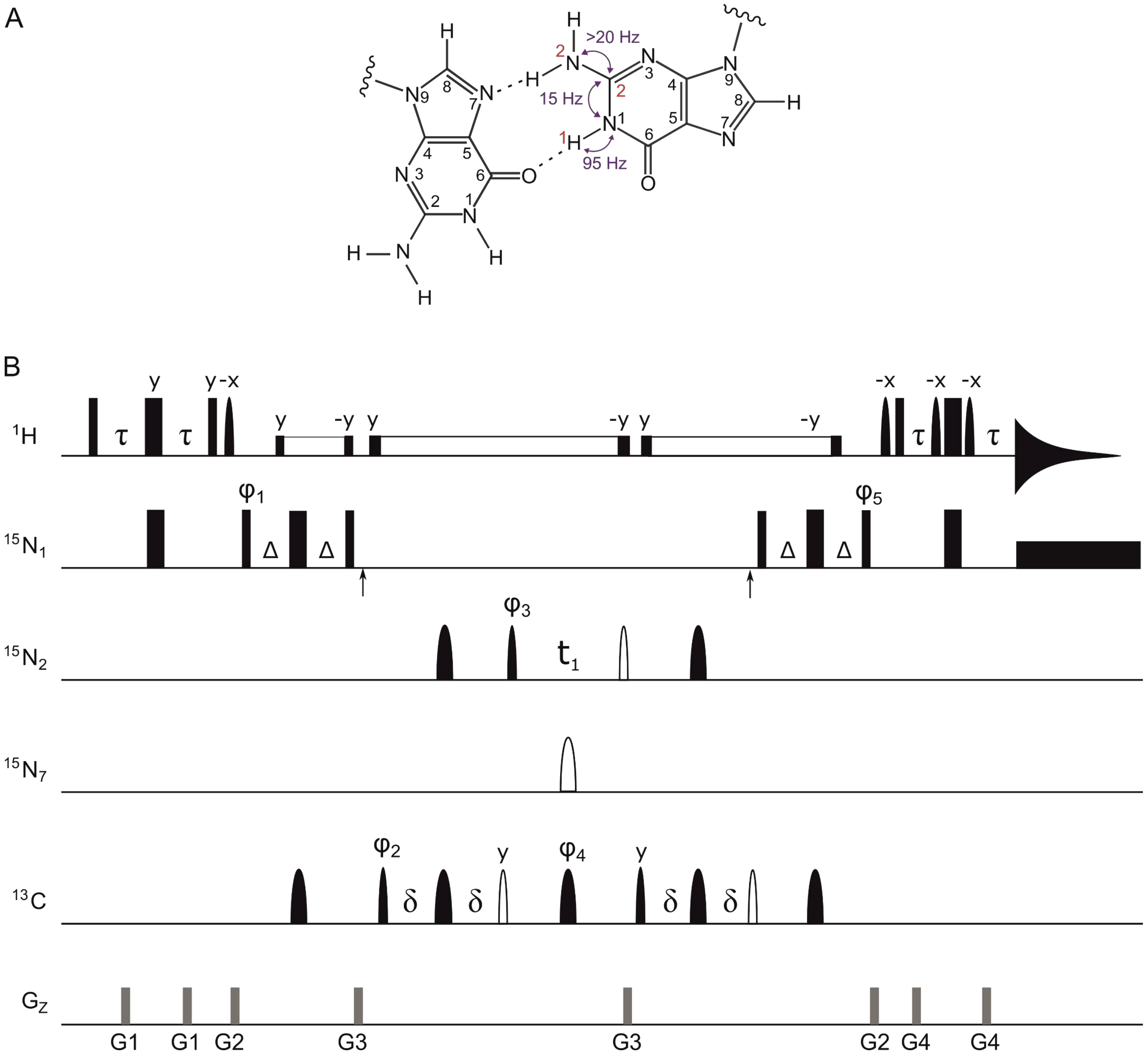

Figure S6. H1(N1C2)N2 pulse sequence and important coupling constants. (A) Schematic representation of the magnetisation transfer in the H1(N1C2)N2 experiment, applicable to ^{13}C,^{15}N-labelled samples. This experiment was previously mentioned in the supporting information of A. Majumdar et al.[32] without giving a pulse sequence. (B) The pulse sequence of the H1(N1C2)N2 experiment correlates the H1 and N2 within a guanine. Narrow (wide) squares and sine-lobes represent 90° (180°) rf-pulses applied along the x-axis unless indicated otherwise. ^{1}H and ^{15}N square pulses are applied at a maximum power of 35.7 kHz and 6.8 kHz, respectively. The ^{1}H and ^{13}C carriers are placed at 4.7 ppm (water) and 155 ppm, respectively. The ^{15}N carrier is positioned at 145 ppm before it is switched to 80 ppm before the 90° pulse of ϕ_2 and is switched back to 145 ppm after the two pulses behind ϕ_3, as indicated by the arrows. The shaped ^{1}H pulses are 1 ms sinc-pulses applied to the water resonance to maintain the water magnetisation along the z-axis during most of the experiment.[25] Filled 180° ^{15}N shaped pulses are 1 ms Gaussian Cascade Q3[28] pulses, while filled and unfilled 90° ^{15}N shaped pulses are 1 ms Gaussian Cascade Q5[28] and time-reversed[29] Q5 pulses, respectively. The unfilled 180° shaped pulse applied to N7 is a phase-modulated 0.9 ms I-BURP-2 pulse[26] applied at 240 ppm. 1.1 kHz

GARP decoupling[30] is applied during acquisition on ^{15}N nuclei. The delays are: τ = 2.8 ms, Δ = 8 ms, and δ = 10 ms. The phase cycles are: ϕ_1 = x,-x, ϕ_2 = 8(x),8(-x), ϕ_3 = 2(x),2(-x), ϕ_4 = 2(x),2(-x), ϕ_5 = 4(x),4(-x), ϕ_{rec} = ABBA with A = x,2(-x),x and B = -x,2(x),-x. Quadrature detection in F1 is achieved by States-TPPI of ϕ_3.[31] All pulsed field gradients have a duration of 1 ms, are of 10 % smoothed square-sine shape, and their strengths are G1 = 30 G/cm, G2 = 20 G/cm, G3 = -5 G/cm, G4 = 40 G/cm.

Spectral quality and assignments

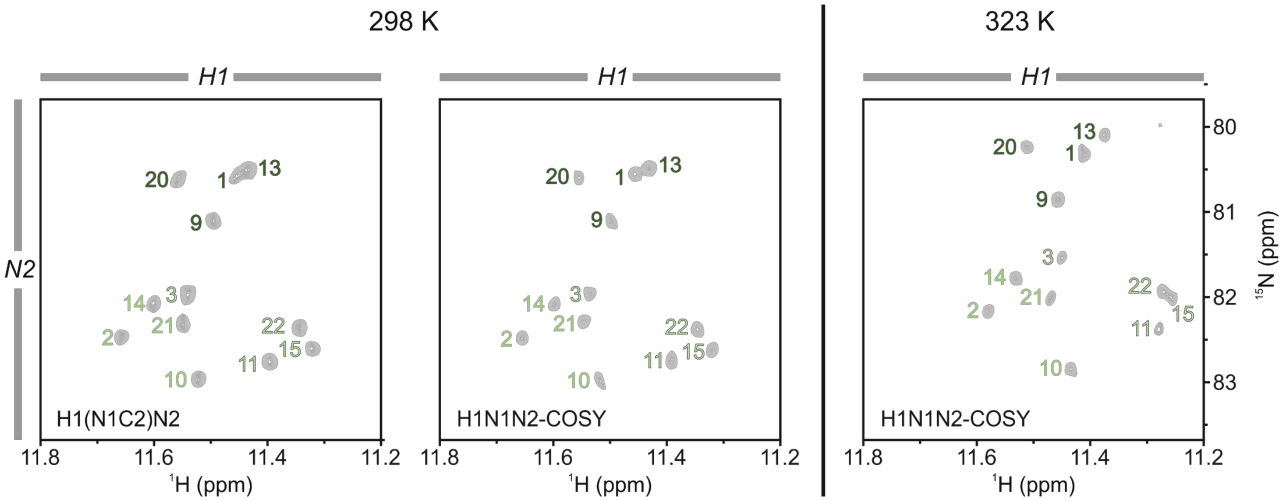

Figure S7. NMR spectra to detect the H1 – N2 correlation via J-coupling. H1(N1C2)N2 at 298 K (left), H1N1N2-COSY at 298 K (middle), and H1N1N2-COSY at 323 K (right) spectra of A6A8U17 (7Q6L) (H_2O/D_2O (9:1), 2 mM KCl, pH 7). The H1(N1C2)N2 spectrum was recorded with a 0.4 mM ^{13}C,^{15}N-G-labelled RNA sample and acquired with a time domain of 2048*70, 16 scans, and 42 min measuring time. The H1N1N2-COSY measurements were recorded with a 0.5 mM ^{15}N-G-labelled RNA sample and acquired with a time domain of 2048*70, 16 scans, and 44 min measuring time. The spectral assignments of the three tetrads are shown with different shades of green.

Methodology for G-quadruplex tetrad assembly

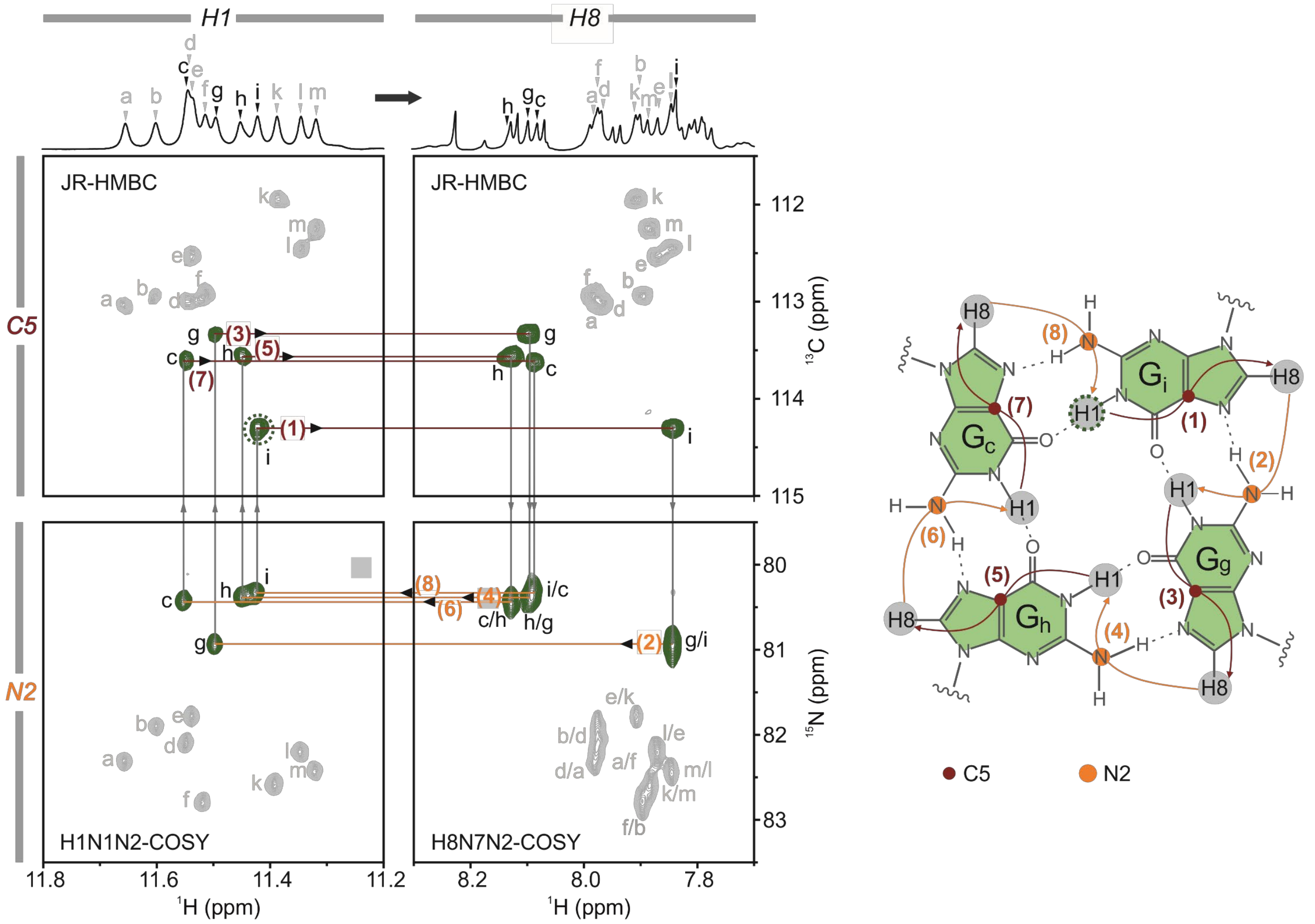

Figure S8. Step-by-step assignment of the G-quadruplex tetrad arrangement. The stepwise assignment of the 5'-G4 tetrad of A6A8U17 (7Q6L) serves as a representative example. The assignment starts within the H1 – C5 region of the JR-HMBC (top left spectrum), whereby the distinct H1 – C5 correlation of G_i (peak in the dotted circle, top right guanine in the schematic G4 tetrad) is chosen as the starting point. Proceeding along the red line (1) to the top right spectrum (H8 – C5 region of the JR-HMBC), the H1 is correlated via the C5 atom to the H8 of the same base. Subsequently, following the grey line from the H8 of G_i in the bottom right spectrum (H8N7N2-COSY) establishes a correlation between H8 and the N2 atom of the neighbouring guanine base G_g. Tracking the orange line (2) to the bottom left spectrum (H1N1N2-COSY) reveals the intraresidue correlation between the N2 and H1 of the second guanine base G_g. Continuing along the grey line in the upper left spectrum, the H1 of G_g is correlated with its C5. The iterative process is repeated for points 3) – 8) until the H1 – C5 correlation of G_i in the dotted circle in the upper left spectrum is reached again. The orange and red lines indicate proton-to-proton correlations, while the grey lines denote the transfer of proton assignment to another spectrum. The JR-HMBC spectrum was recorded with an RNA sample (NA) at 1.3 mM, and the HNN-COSYs were acquired with a 0.45 mM ^{15}N-G-labelled RNA sample (H_2O/D_2O (9:1), 2 mM KCl, pH 7, 298 K).

Exchange experiments

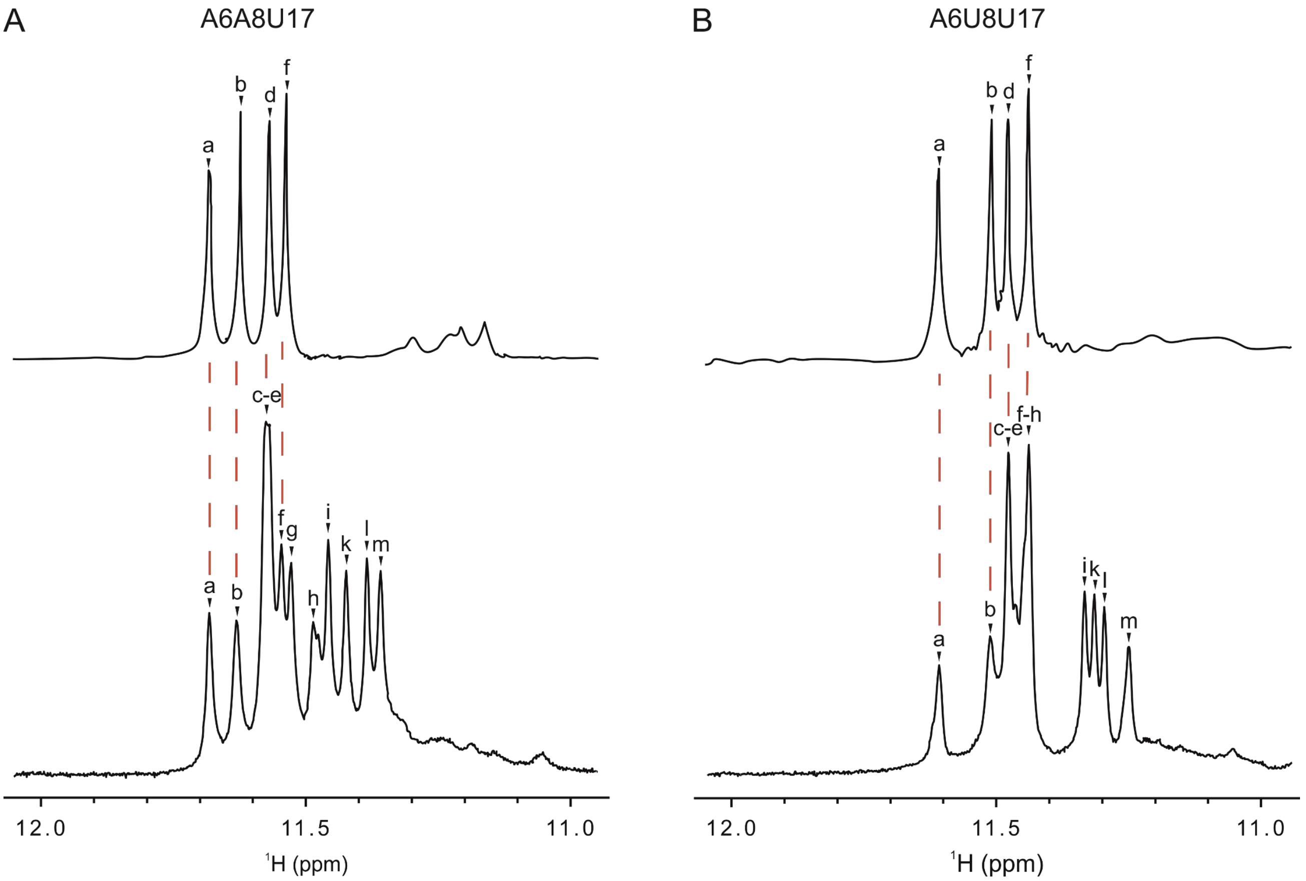

Figure S9. ^{1}H NMR exchange experiments. A) 1D ^{1}H NMR spectra of the imino-proton region of A6A8U17 (7Q6L) in H_2O/D_2O (9:1) (bottom) and after lyophilising and re-dissolving the sample in D_2O without re-annealing the sample (top). B) 1D ^{1}H NMR spectra of the imino-proton region of A6U8U17 (7Q48) in H_2O/D_2O (9:1) (bottom) and after lyophilising and re-dissolving the sample in D_2O without re-annealing the sample (top). The four H1 imino proton signals that belong to the guanines of the centre tetrad of the G4s (d–b–f–a; red dashed lines) are preserved, confirming the monomeric state. In a dimer, the signals from the two stacking cores (for the 5' tetrad: c–i–g–h) would also be protected from deuterium exchange and, therefore, visible in the top 1D ^{1}H NMR spectra. All spectra were recorded in 2 mM KCl at pH 7 (pD 7) at 298 K using 1.3 mM (NA) A6A8U17 and 1 mM (NA) A6U8U17, respectively.

Tetrads assembly and sequential assignment of the G4 core by NOE correlations

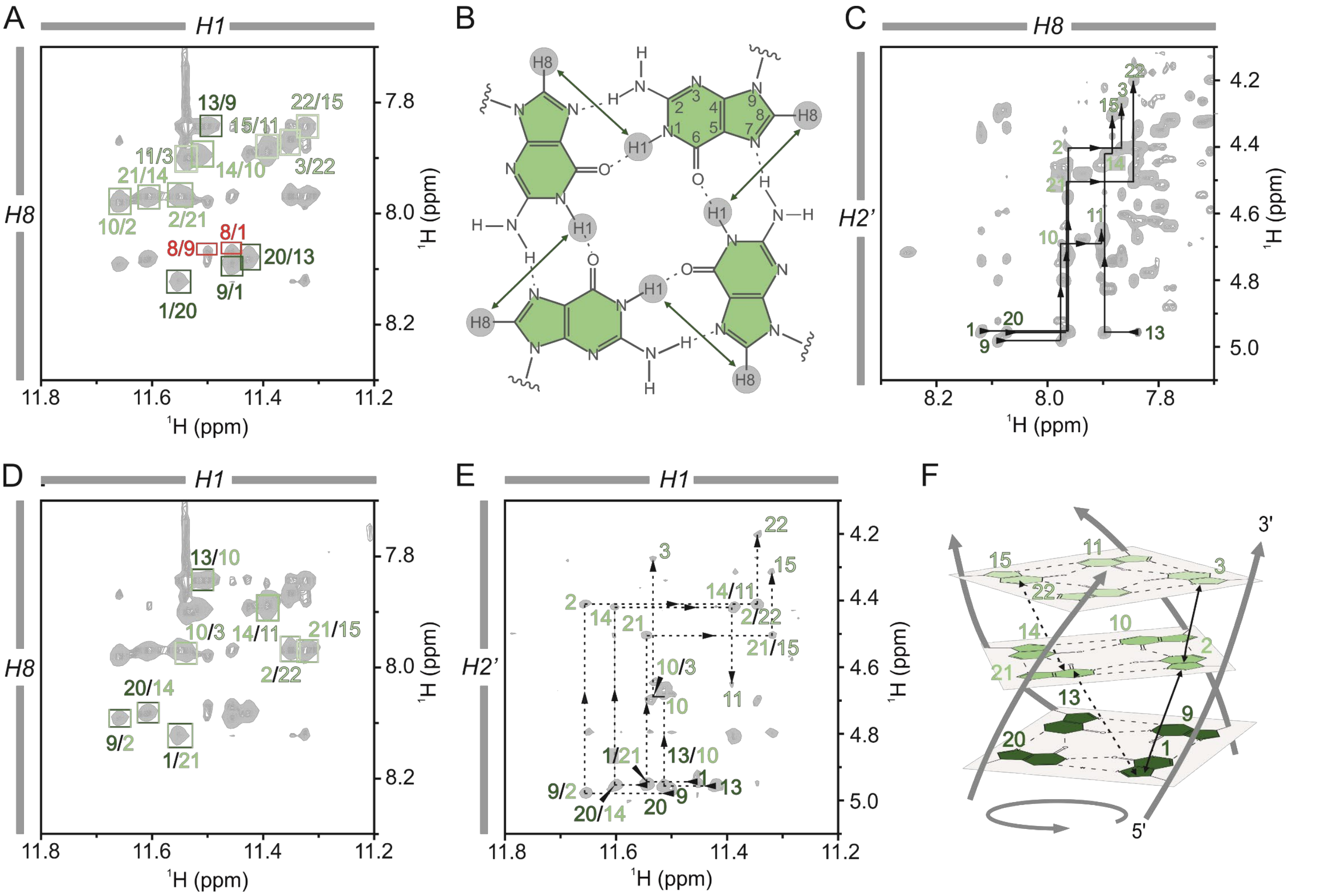

Figure S10. Tetrad assembly and sequential G-quadruplex assignment. A) The H1 – H8 region of the [^{1}H,^{1}H]-NOESY spectrum of A6A8U17 (7Q6L) recorded in H_2O/D_2O (9:1) with the intraplanar through-space H1 – H8 correlations of the guanines assigned. Each tetrad is indicated in a different shade of green. The correlations marked in red correspond to correlations of the H2 of the adenine A8 with the H1 of the guanines G1 and G9, indicating the stacking interaction of A8 below the 5'-tetrad. B) Schematic presentation of a G4 tetrad with the intraplanar H1 – H8 NOE correlations. C) The H8 – H2' region of the [^{1}H,^{1}H]-NOESY spectrum of A6A8U17 (7Q6L) recorded in D_2O with the typical sequential correlations (indicated by solid black lines) from the sugar H2' to the H8 of the following guanine of the same strand from which the four G-strands of the core can be determined. D) The same H1 – H8 region of the [^{1}H,^{1}H]-NOESY as in A but with the lower-intensity, interplanar through-space H1 – H8 correlations of the guanines assigned. The higher-intensity signals correspond to intraplanar correlations (assigned in A). E) H1 – H2' region of the [^{1}H,^{1}H]-NOESY spectrum of A6A8U17 (7Q6L) recorded in H_2O, showing interplanar correlations between sugar H2' and H1 of the stacking guanine from the left neighbouring strand (indicated by dashed black lines). This 5' to 3' directed interplanar pattern $G_1 \rightarrow G_{21} \rightarrow G_{15}$, $G_9 \rightarrow G_2 \rightarrow G_{22}$, $G_{13} \rightarrow G_{10} \rightarrow G_3$ and $G_{20} \rightarrow G_{14} \rightarrow G_{11}$ illustrates that G4s form right-handed helices. F) Schematic representation of the right-handed helical core with the colour-coded tetrads and the labelled guanines. Sequential correlations, as shown in C, and interplanar correlations, as shown in E, are represented by black solid lines for the $G_1 – G_2 – G_3$ strand and by black dashed lines for the $G_1 – G_{21} – G_{15}$ correlations. The [^{1}H,^{1}H]-NOESY spectra were recorded using a 1.3 mM (NA) A6A8U17 in 2 mM KCl at pH 7 (pD 7) and 298 K. All correlations are assigned so that the first nucleotide number corresponds to the F2 resonance (horizontal axis) and the second to the F1 resonance (vertical axis).

Sequential Assignment

A6A8U17: 5' - GGGCC AUAGG GUGGG AUCUG GG - 3'
1 6 11 16 21

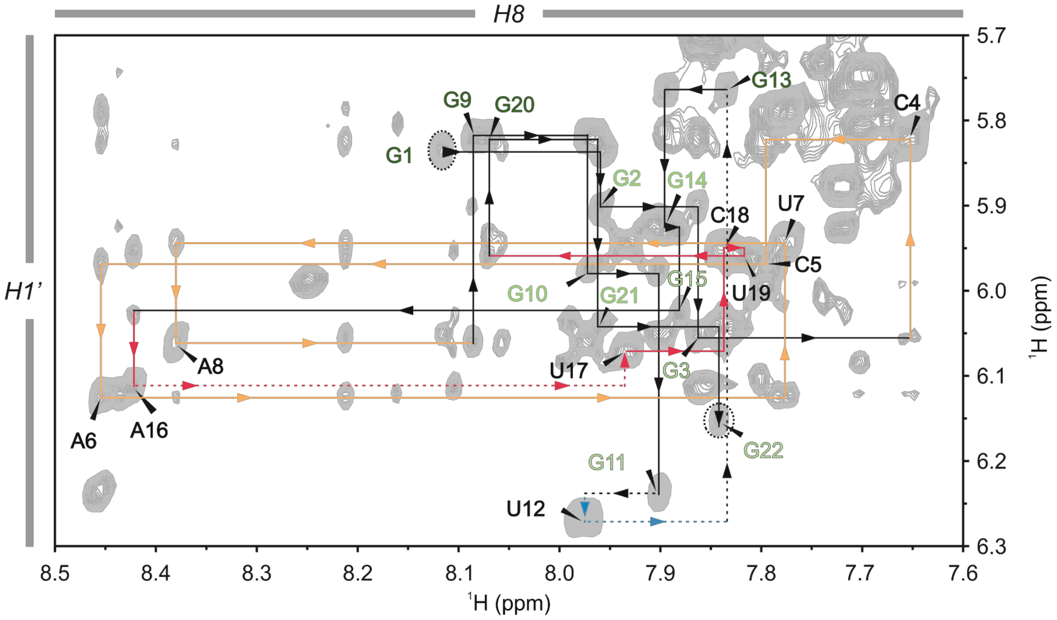

Figure S11. Complete assignment of the sequential walk region. The H6/H8 – H1' region of the NOESY spectrum of A6A8U17 (1.3 mM (NA) RNA, 100% D_2O, 2 mM KCl, pD 7, 298 K) shows the sequential correlations through the guanine stacks as black lines and the loops' sequential correlation as orange, blue and red lines, respectively. The solid lines mark regions where the sequential path can be followed by H6/H8 – H1' correlations, while the dashed lines mark regions where these correlations are absent. Guanines that belong to the same tetrad are coloured the same shade of green.

Glycosidic bond orientation

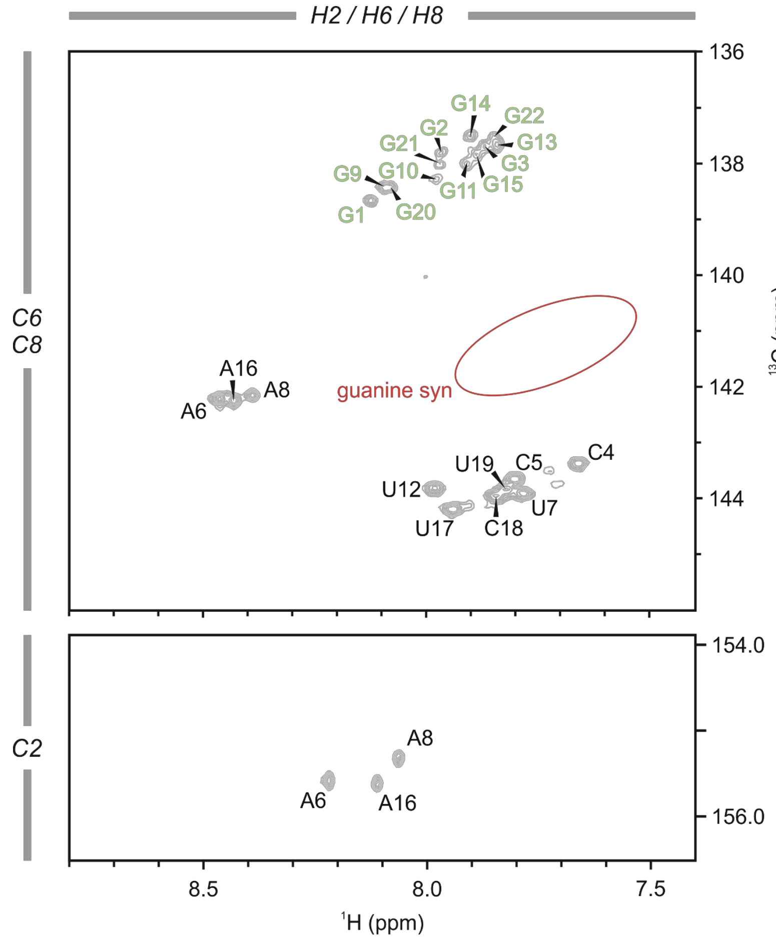

Figure S12. Glycosidic bond orientation of the guanines. Aromatic region of the [^{13}C,^{1}H]-HSQC spectrum of A6A8U17 (7Q6L) showing the H2 – C2, H6 – C6, and H8 – C8 correlations with guanine residues marked in green. The red ellipse indicates the chemical shift region characteristic for *syn*-guanines. The absence of signals in this region confirms that all guanines adopt the *anti*-conformation. The spectrum was recorded in 100% D_2O, 2 mM KCl, at pD 7 and 298 K, with an RNA (NA) concentration of 1.3 mM.

Sugar conformation

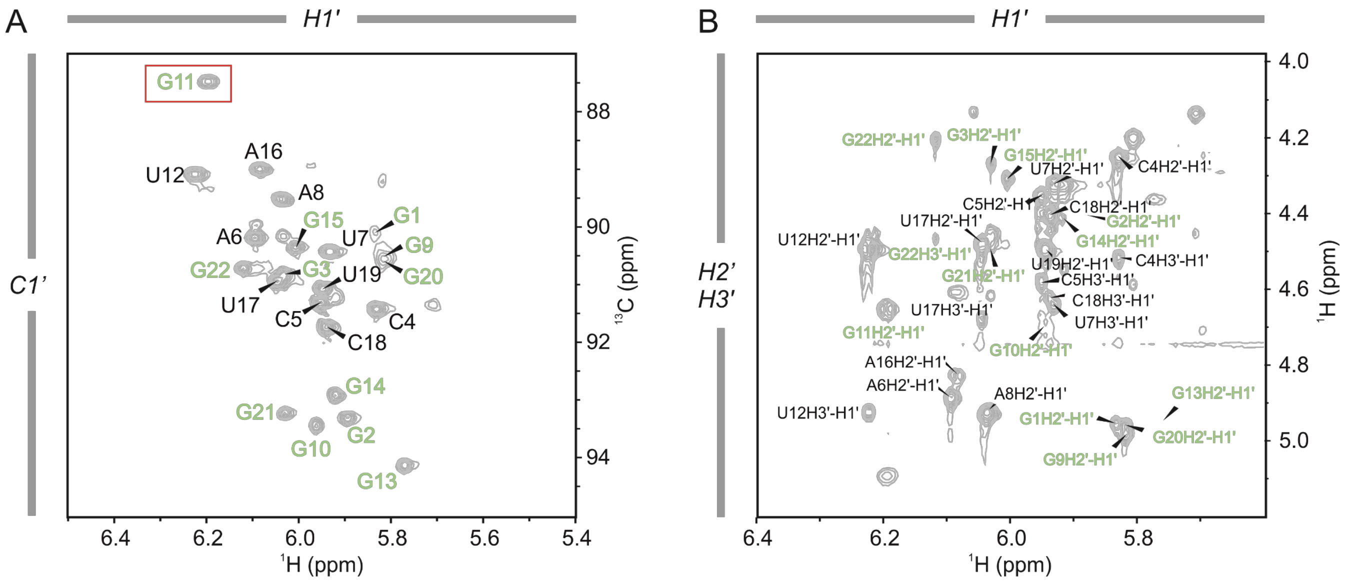

Figure S13. Determination of the sugar conformations. (A) The H1’ – C1’ region of the [^{13}C,^{1}H]-HSQC and (B) the H1’ – H2’/H3’ region of the [^{1}H,^{1}H]-TOCSY provide insight into the sugar conformations of the nucleotides in A6A8U17 (7Q6L). Guanine residues are marked in green. A pronounced upfield shift of the C1’ resonance for G11 (red box) indicates its adoption of the C2’-endo sugar conformation, while all other guanines exhibit the C3’-endo sugar conformation. The H1’ – H2’ and H1’ – H3’ peak intensities in the TOCSY spectrum further confirm the sugar conformations, with strong intensity peaks corresponding to the C2’-endo conformation. Both spectra were recorded in 100% D_2O, 2 mM KCl, at pD 7 and 298 K, with an RNA (NA) concentration of 1.3 mM.

NOE assignment of the G-quadruplex core

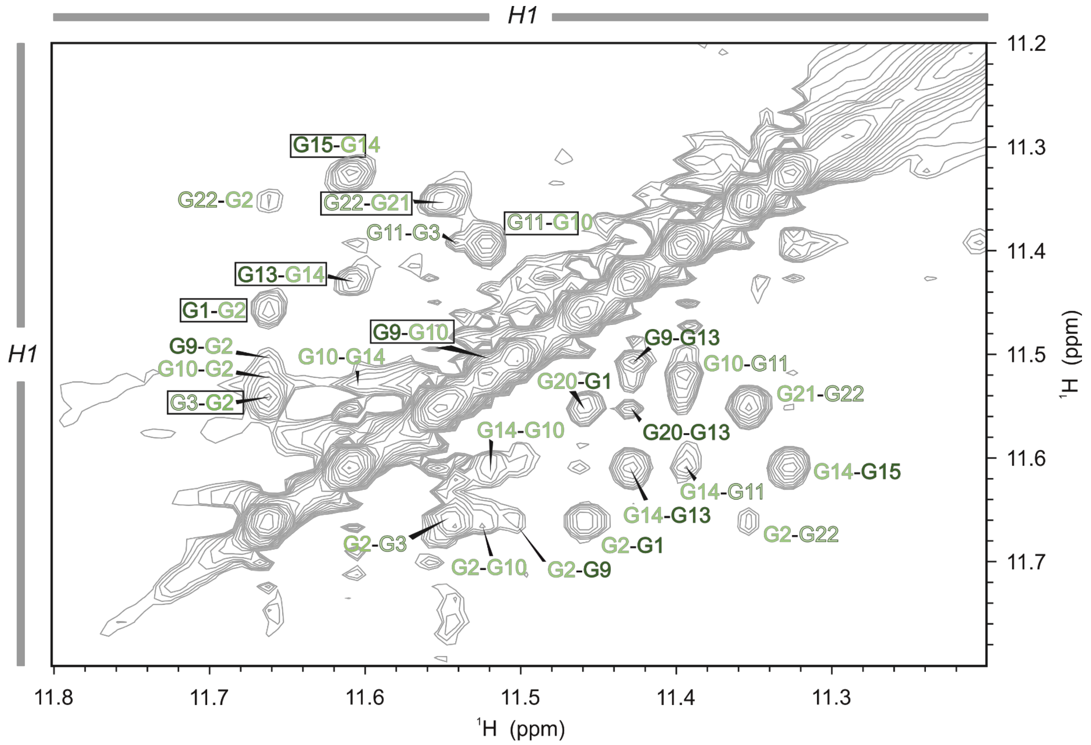

Figure S14. H1 – H1 NOE assignment in the G-quadruplex core. The H1 – H1 region of the [^{1}H,^{1}H]-NOESY spectrum of A6A8U17 (7Q6L) reveals intraplanar and interplanar H1-H1 correlations within the G-quadruplex core. Guanines are coloured based on their respective tetrad: the 5'-end tetrad is shown in dark green, the middle tetrad in green, and the 3'-end tetrad in light green. Black boxes mark sequential H1 – H1 NOEs above the diagonal. The spectrum was recorded in H_2O/D_2O (9:1), 2 mM KCl, at pD 7 and 298 K, with an RNA concentration of 1.3 mM.

Calculated BCL2 structures

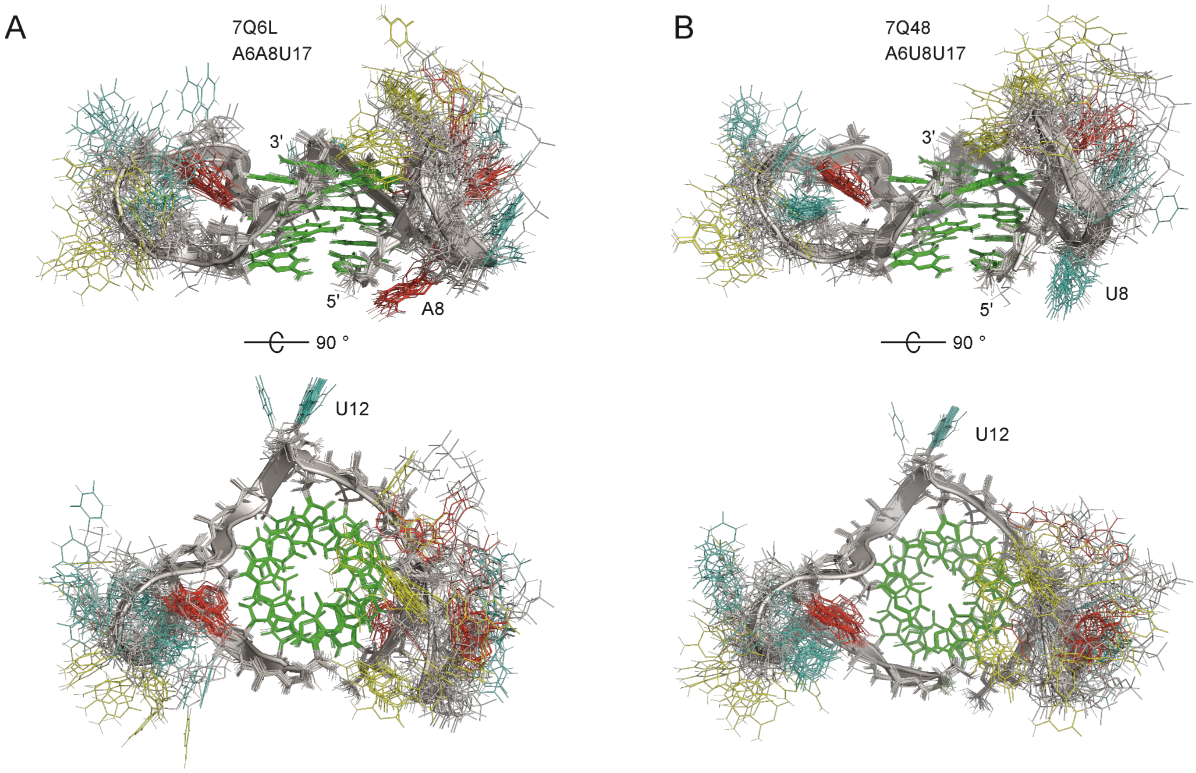

Figure S15. Overlay of the calculated *BCL2* structures. Complete top and side view of the overlay of the 20 lowest-energy structures of 7Q6L and 7Q48, respectively. Backbone and ribose sugars are depicted in grey, guanines in green, adenines in red, uracils in cyan, and cytosines in yellow.

Area and planarity of the BCL2 G-quadruplex tetrads

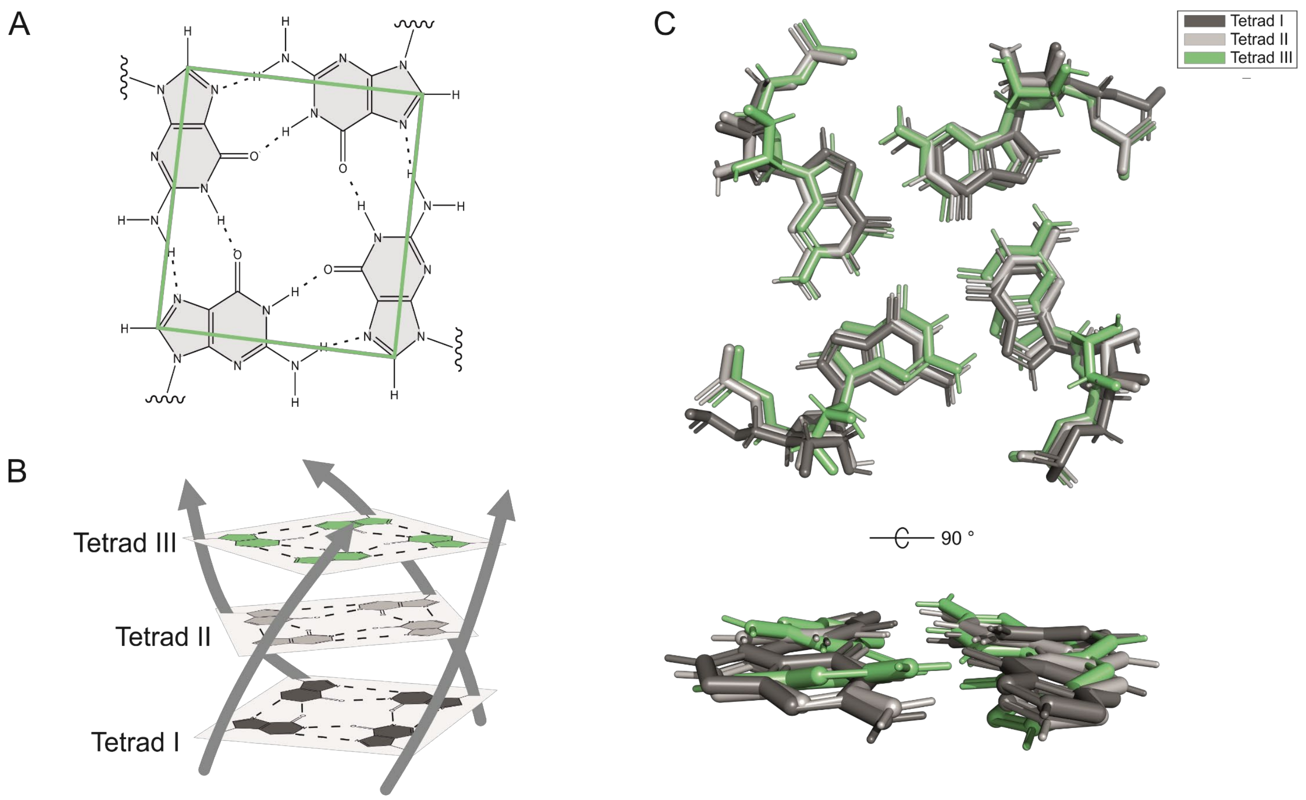

Figure S16. Comparison of the tetrad areas within the G-quadruplex A) Schematic of a G4 tetrad showing the tetrad area used in the calculations, which is defined by the C8 atoms of the guanines. B) Schematic representation of a parallel core with the designation of the tetrads. C) Top and side view of the superposition of the three tetrads of A6A8U17 (7Q6L), with the 3'-end tetrad (III) shown in green and the other two in two shades of grey. This overlay shows that tetrad III has a distinct smaller area than tetrads I and II.

Schematic structures of all analysed G-quadruplexes

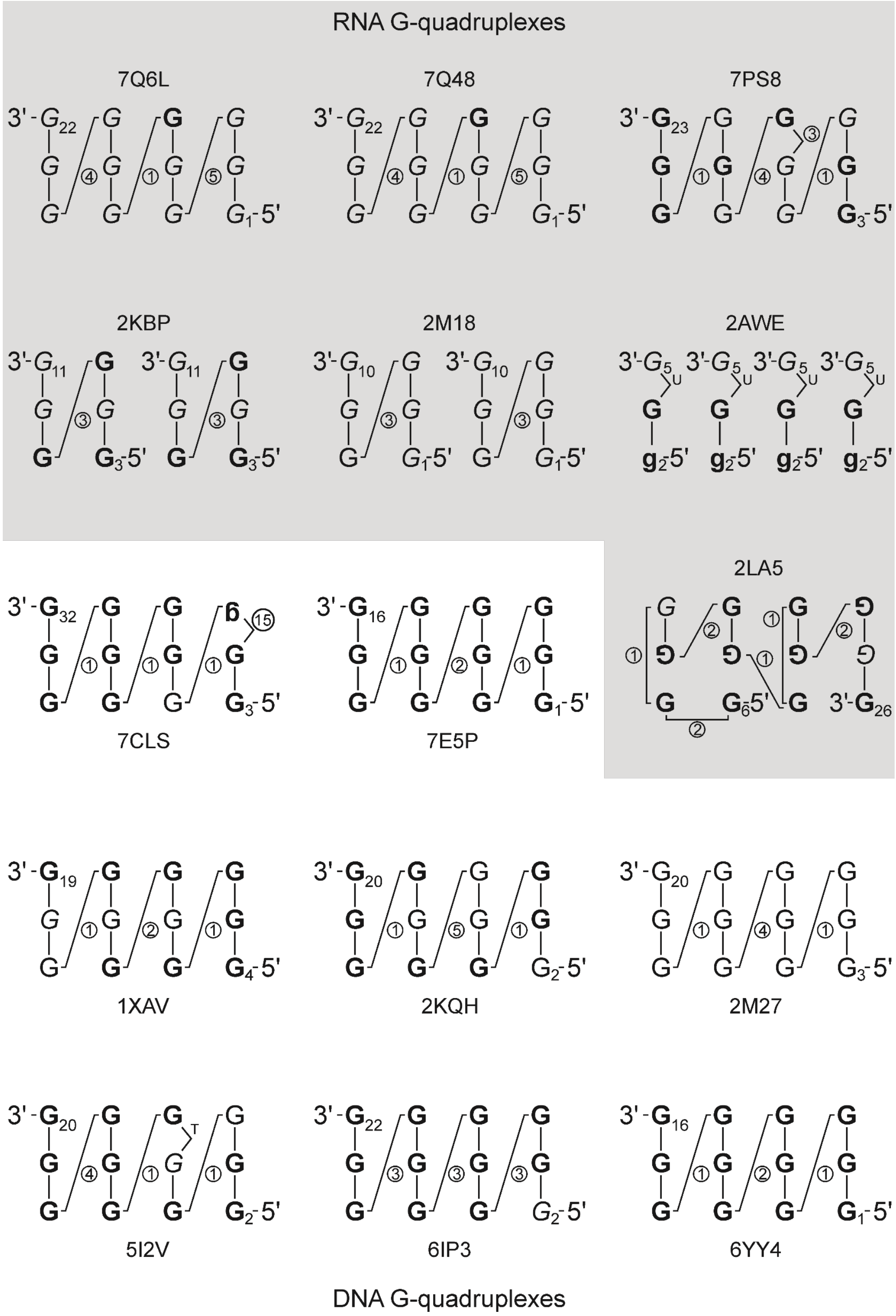

Figure S17. Schematic depiction of the core structures of all analysed G-quadruplexes. For this scheme, the graphical shorthand for G4s proposed by Banco and Ferré-D'Amaré[33] was used in which only the core guanines are shown. Bold guanines represent C2'-endo sugar conformation, italic guanines are in C3'-endo sugar conformation, and normal letter guanines show other sugar puckers. Upper- and lower-case guanines stand for *anti* and *syn* conformation, respectively. Inverted guanines indicate a reversed strand polarity compared to the rest of the G4. The loop lengths are displayed within circles, and bulges are marked by >. 7Q6L, 7Q48, 2KBP, 2M18, 7E5P, 1XAV, 2M27, 2KQH, 6IP3, 6YY4 are canonical and 7PS8, 2AWE, 2LA5, 7CLS, 5I2V are non-canonical G4 structures.

Overview of all calculated NMR G-quadruplex structures

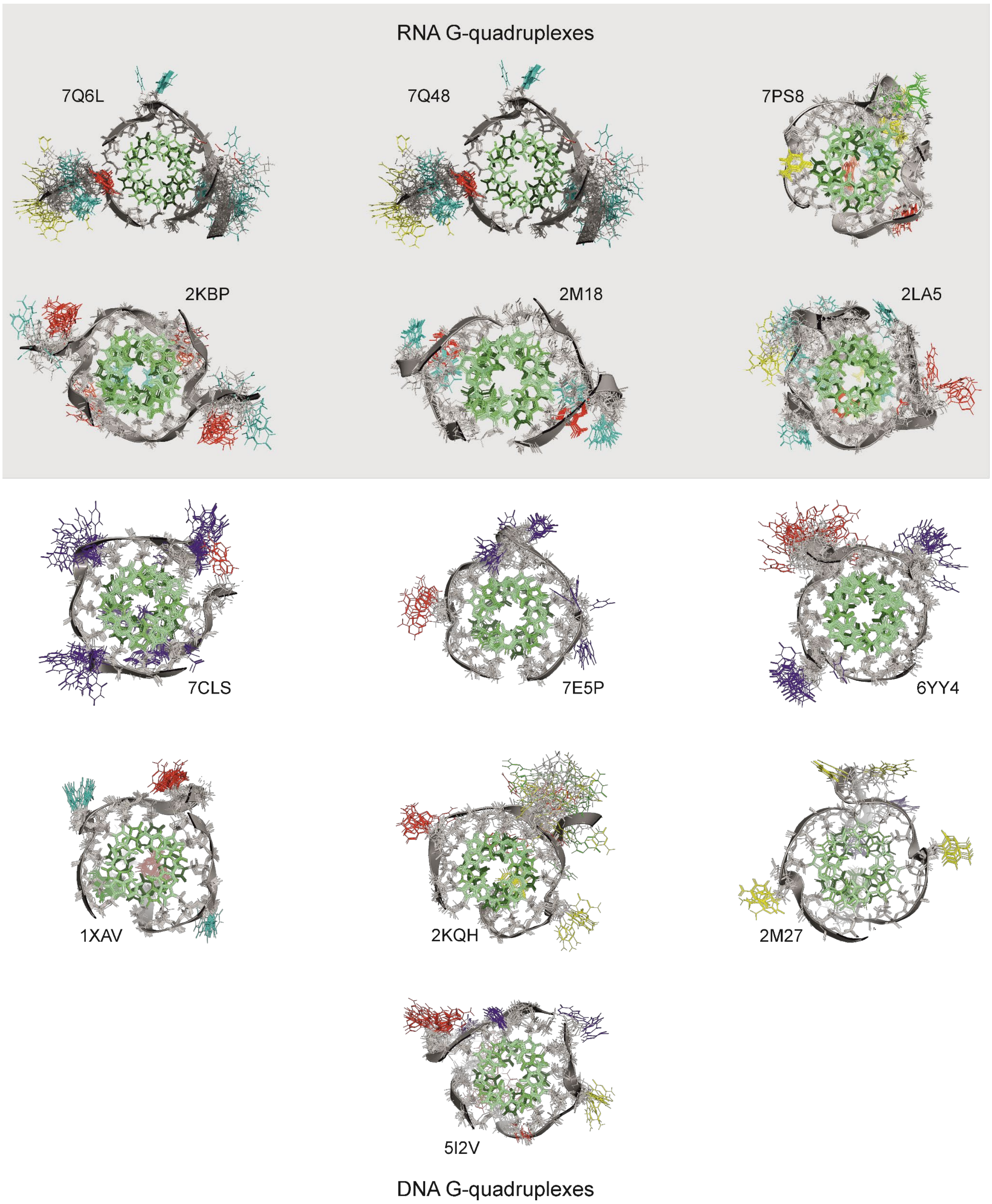

Figure S18. Overview of all calculated NMR G-quadruplex structures. The ten lowest-energy structures from the 13 calculated NMR G-quadruplex structures were aligned based on their G-quadruplex cores, emphasizing the high-definition nature of this central region. To enhance visualization, nucleotides above and below the G-quadruplex core were clipped. The corresponding PDB codes are displayed next to each structure. The colour scheme is as follows: backbone and sugar in grey; bases: guanines in green, adenines in red, cytosines in yellow, uracils in cyan, and thymines in blue.

Comparison of geometrical G4 core parameters

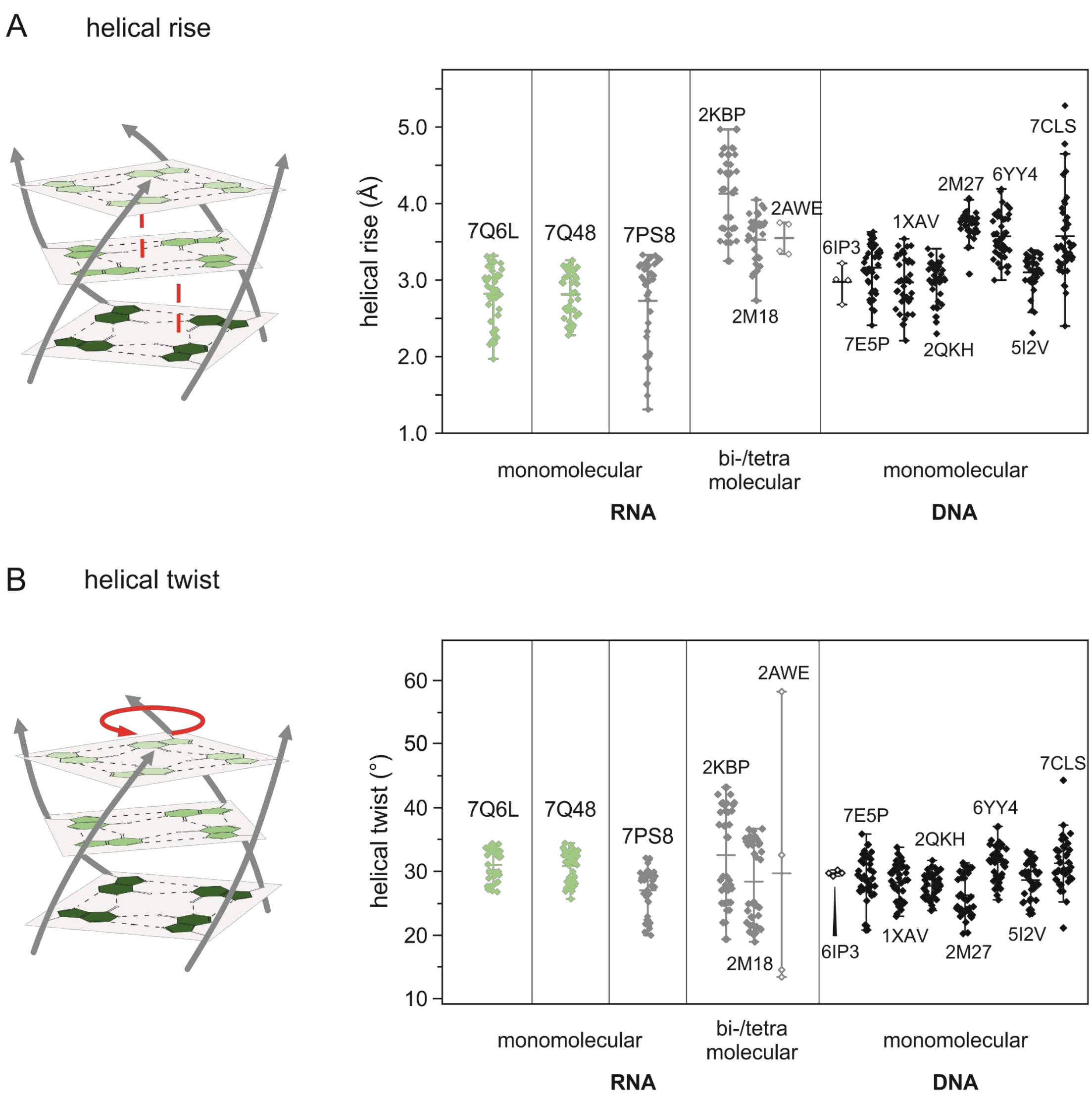

Figure S19. Comparison of the helical rise and helical twist of G4 tetrads. A) Schematic representation of the helical rise in the core (left) and a jitter plot of the helical rises (right) of the two BCL2 mutants A6A8U17 (7Q6L) and A6U8U17 (7Q48) in comparison to the other monomolecular RNA-G4, the bi- and tetramolecular RNA-G4s and the monomeric, parallel DNA-G4s. Crystal structures are depicted with white diamonds, while NMR structures are coloured diamonds (green, grey, black). The PDB codes of all structures are shown in the plot. B) Schematic representation of the helical twist in the core (left) and jitter plot of the helical twists (right) for the same structures as mentioned in A.

NMR restraints and structural statistics

Table S2. NMR restraints and structural statistics for the two *BCL2* G-quadruplex mutants. All statistics are given for the respective 20 lowest energy structures out of 200 calculated structures.

	**A6A8U17 (7Q6L)**		**A6U8U17 (7Q48)**	
	Non-exchangeable	Exchangeable	Non-exchangeable	Exchangeable
Total NOE restraints	273	98	241	92
Intraresidual	136	22	135	21
Sequential (|*i*-*j*| = *1*)	117	22	89	22
Long range (|*i*-*j*| ≥ *2*)	20	54	17	49
NOE restraints per residue	16.9		15.1	
per core residue	18.2		17.5	
per loop residue	15.5		12.7	
NOE violations > 0.2 Å	0		0	
Dihedral restraints	68		70	
Dihedral violations > 5°	0		0	
Hydrogen bond	48		48	
RMSD				
overall	3.29 ± 1.03 Å		3.02 ± 0.68 Å	
core	0.60 ± 0.19 Å		0.54 ± 0.17 Å	

Planarity

Table S3. Overview of the tetrad planarities of all analysed RNA-G quadruplexes. The planarity value for the NMR structures is the mean value of the 10 structures with the lowest numbers of geometric issues, and the error corresponds to σ, while there is only one value for the crystal structure.

**RNA G4**							
**PDB**	NMR A6U8U17 (7Q48)	NMR A6A8U17 (7Q6L)	NMR Monomol. 7PS8	NMR G4-duplex 2LA5	NMR Bimol. 2KBP	NMR Bimol. 2M18	Crystal Tetramol. 2AWE
Tetrad I	0.33±0.02	0.32±0.03	0.31±0.02	0.19±0.01	0.22±0.03	0.34±0.06	0.176
Tetrad II	0.24±0.02	0.23±0.02	0.31±0.04	0.21±0.03	0.20±0.04	0.34±0.06	0.259
Tetrad III	0.42±0.03	0.42±0.03	0.26±0.06	0.13±0.03	0.22±0.03	0.45±0.05	0.291

Table S4. Overview of the tetrad planarities of all analysed DNA-G quadruplexes. The planarity value for the NMR structures is the mean value of the 10 structures with the lowest numbers of geometric issues, and the error corresponds to σ, while there is only one value for the crystal structure.

**DNA G4 (monomolecular, parallel)**								
**PDB**	NMR 5I2V	NMR 7E5P	NMR 1XAV	NMR 2KQH	NMR 2M27	NMR 6YY4	NMR G4-duplex 7CLS	Crystal 6IP3
Tetrad I	0.21±0.08	0.19±0.05	0.22±0.05	0.26±0.08	0.07±0.01	0.04±0.02	0.12±0.03	0.126
Tetrad II	0.18±0.05	0.13±0.03	0.23±0.04	0.30±0.10	0.05±0.02	0.06±0.01	0.12±0.05	0.212
Tetrad III	0.19±0.05	0.18±0.03	0.17±0.05	0.38±0.12	0.10±0.01	0.03±0.01	0.11±0.03	0.338

References

1 C. Kao, S. Rüdisser and M. Zheng, *Methods*, 2001, **23**, 201–205.
2 S. Gallo, M. Furler and R. K. O. Sigel, *CHIMIA*, 2005, **59**, 812–816.
3 Z. Wang*, et al.,* 7Q6L, https://www.rcsb.org/.
4 Z. Wang, et al., 7Q48, https://www.rcsb.org/.
5 J.-L. Mergny and L. Lacroix, *Curr. Protoc. Nucleic Acid Chem.*, 2009, **37**, 17.1.1-17.1.15.
6 A. T. Phan, *J. Biomol. NMR*, 2000, **16**, 175–178.
7 A. Majumdar, A. Kettani and E. Skripkin, *J. Biomol. NMR*, 1999, **14**, 67–70.
8 A. T. Phan, *J. Magn. Reson.*, 2001, **153**, 223–226.
9 G. W. Vuister and A. Bax, *J. Magn. Reson.*, 1992, **98**, 428–435.
10 K. Hallenga and G. M. Lippens, *J. Biomol. NMR*, 1995, **5**, 59–66.
11 J. L. Markley*, et al.*, *J. Biomol. NMR*, 1998, **12**, 1–23.
12 W. Lee, M. Tonelli and J. L. Markley, *Bioinformatics*, 2014, **31**, 1325–1327.
13 P. Güntert, C. Mumenthaler and K. Wüthrich, *J. Mol. Biol.*, 1997, **273**, 283–298.
14 C. D. Schwieters*, et al.*, *J. Magn. Reson.*, 2003, **160**, 65–73.
15 C. D. Schwieters, J. J. Kuszewski and G. M. Clore, *Prog. Nucl. Magn. Reson. Spectrosc.*, 2006, **48**, 47–62.
16 G. A. Bermejo and C. D. Schwieters, *Methods Mol. Biol.*, 2018, **1688**, 311–340.
17 G. A. Bermejo, G. M. Clore and C. D. Schwieters, *Structure*, 2016, **24**, 806–815.
18 L. L. Schrödinger, *The PyMOL Molecular Graphics System, Version 1.8*, 2015.
19 X.-J. Lu, *Nucleic Acids Res.*, 2020, **48**, e74.
20 X.-J. Lu, H. J. Bussemaker and W. K. Olson, *Nucleic Acids Res.*, 2015, **43**, e142.
21 The MathWorks Inc., *MATLAB Version: 9.14.0 (R2023a)*, The MathWorks Inc., Natick, Massachusetts, United States, 2023.
22 D.-H. Zhang*, et al.*, *Biochem.*, 2010, **49**, 4554–4563.
23 R. Shahid, A. Bugaut and S. Balasubramanian, *Biochem.*, 2010, **49**, 8300–8306.
24 P. Büchner, W. Maurer and H. Rüterjans, *J. Magn. Reson.*, 1978, **29**, 45–63.
25 R. Freeman, *Chem. Rev.*, 1991, **91**, 1397–1412.
26 H. Geen and R. Freeman, *J. Magn. Reson.*, 1991, **93**, 93–141.
27 E. Kupce, J. Boyd and I. D. Campbell, *J. Magn. Reson.*, 1995, **106**, 300–303.
28 L. Emsley and G. Bodenhausen, *J. Magn. Reson.*, 1992, **97**, 135–148.
29 J. Cavanagh, *Protein NMR spectroscopy. Principles and practice*, Academic Press, Amsterdam, Boston, 2nd edn., 2007.
30 A. Shaka, P. Barker and R. Freeman, *J. Magn. Reson.*, 1985, **64**, 547–552.
31 D. Marion*, et al.*, *J. Magn. Reson.*, 1989, **85**, 393–399.
32 A. Majumdar*, et al.*, *J. Biomol. NMR*, 1999, **15**, 207–211.
33 M. T. Banco and A. R. Ferré-D'Amaré, *RNA*, 2021, **27**, 390–402.
